# Synergy principle of single active centers and microenvironment for Cr-MFI-catalyzed alkane dehydrogenation

**DOI:** 10.1093/nsr/nwaf405

**Published:** 2025-09-22

**Authors:** Zhong-Pan Hu, Gangqiang Qin, Jingfeng Han, Yijun Zheng, Zhen Liu, Yong Jiang, Xiaozhi Su, Te Ji, Min Li, Zhong-Yong Yuan, Jianping Xiao, Svetlana Mintova, Yingxu Wei, Zhongmin Liu

**Affiliations:** National Engineering Research Center of Lower-Carbon Catalysis Technology, Dalian National Laboratory for Clean Energy, Collaborative Innovation Center of Chemistry for Energy Materials (iChEM), Dalian Institute of Chemical Physics, Chinese Academy of Sciences, Dalian 116023, China; State Key Laboratory of Catalysis, Dalian Institute of Chemical Physics, Chinese Academy of Sciences, Dalian 116023, China; University of Chinese Academy of Sciences, Beijing 100049, China; National Engineering Research Center of Lower-Carbon Catalysis Technology, Dalian National Laboratory for Clean Energy, Collaborative Innovation Center of Chemistry for Energy Materials (iChEM), Dalian Institute of Chemical Physics, Chinese Academy of Sciences, Dalian 116023, China; National Engineering Research Center of Lower-Carbon Catalysis Technology, Dalian National Laboratory for Clean Energy, Collaborative Innovation Center of Chemistry for Energy Materials (iChEM), Dalian Institute of Chemical Physics, Chinese Academy of Sciences, Dalian 116023, China; Shanghai Synchrotron Radiation Facility (SSRF), Shanghai Institute of Applied Physics, Chinese Academy of Sciences, Shanghai 201800, China; Shanghai Synchrotron Radiation Facility (SSRF), Shanghai Institute of Applied Physics, Chinese Academy of Sciences, Shanghai 201800, China; Shanghai Synchrotron Radiation Facility (SSRF), Shanghai Institute of Applied Physics, Chinese Academy of Sciences, Shanghai 201800, China; Shanghai Synchrotron Radiation Facility (SSRF), Shanghai Institute of Applied Physics, Chinese Academy of Sciences, Shanghai 201800, China; Elettra–Sincrotrone Trieste, Trieste 34149, Italy; School of Materials Science and Engineering, Nankai University, Tianjin 300350, China; State Key Laboratory of Catalysis, Dalian Institute of Chemical Physics, Chinese Academy of Sciences, Dalian 116023, China; University of Chinese Academy of Sciences, Beijing 100049, China; Normandie University, Laboratory of Catalysis and Spectrochemistry (LCS), ENSICAEN, UNICAEN, CNRS, Caen 14050, France; National Engineering Research Center of Lower-Carbon Catalysis Technology, Dalian National Laboratory for Clean Energy, Collaborative Innovation Center of Chemistry for Energy Materials (iChEM), Dalian Institute of Chemical Physics, Chinese Academy of Sciences, Dalian 116023, China; National Engineering Research Center of Lower-Carbon Catalysis Technology, Dalian National Laboratory for Clean Energy, Collaborative Innovation Center of Chemistry for Energy Materials (iChEM), Dalian Institute of Chemical Physics, Chinese Academy of Sciences, Dalian 116023, China; State Key Laboratory of Catalysis, Dalian Institute of Chemical Physics, Chinese Academy of Sciences, Dalian 116023, China; University of Chinese Academy of Sciences, Beijing 100049, China

**Keywords:** propane dehydrogenation, metal zeolite, single Cr center, zeolite microenvironment, dehydrogenation mechanism

## Abstract

Propane dehydrogenation (PDH) to propylene holds immense industrial application value and encompasses pivotal scientific issues in fossil resources utilization, particularly the C–H bond activation and transformation in alkane conversion. Metal-containing zeolites have emerged as efficient catalysts for alkane dehydrogenation. However, the fundamental understanding of how the metal center and zeolite microenvironment participate in alkane dehydrogenation remains elusive. Here we constructed a Cr-MFI zeolite featuring single Cr centers embedded within the MFI framework and utilized this highly efficient PDH catalyst to comprehensively illustrate the synergistic interplay between metal active centers and the zeolite microenvironment for alkane dehydrogenation. Through *in situ* X-ray absorption spectroscopy (XAS) and *in situ* Fourier transform infrared (FTIR) spectroscopy, we successfully captured the dynamic evolution of Cr electronic states and the migration of H species to Cr and its adjacent O atoms under PDH conditions. Theoretical calculations and isotope labeling elucidated the synergy principle between Cr active centers and the zeolite microenvironment in Cr-MFI, demonstrating that the zeolite microenvironment intensifies propane activation and the flexible Cr–O–Si centers consecutively extract H* from propane. These findings provide great insights into the dynamic catalytic behavior of metal-zeolite systems under alkane dehydrogenation conditions and offer valuable guidelines for the rational design and optimization of such catalysts for industrial application.

## Teaser

This study reveals how Cr active sites and the zeolite microenvironment in Cr-MFI synergistically enhance alkane dehydrogenation, with the zeolite framework promoting alkane activation while Cr centers sequentially extract H* species.

## INTRODUCTION

**Figure 1.**
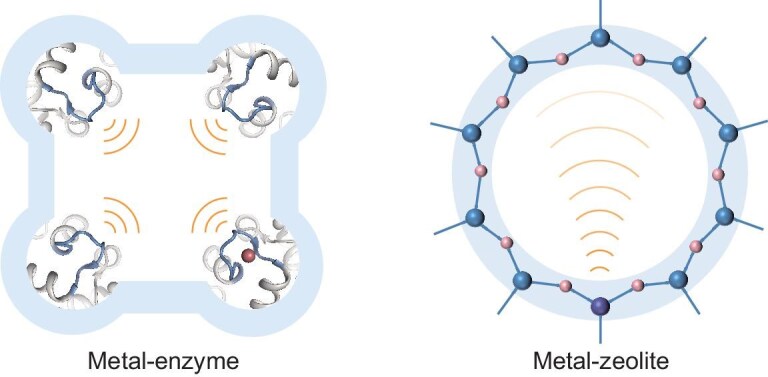
Schematic illustration of the reinforced catalysis from dynamical interactions between the active site and the surrounding microenvironment in metalloenzyme and metal-zeolite materials. The metalloenzyme contains dynamic and flexible tunnels (electric field orientation from metal and organic fragment grafting to the tunnels). The metal-zeolite contains well-defined topological structure and pores (confined electric field orientation from metal to the whole pore environment). Both of these two kinds of materials are a combination of active sites and microenvironments, with similar features of homogeneity, cooperation and periodicity.

The cooperation between the microenvironment and active sites in zeolite catalysts produces a unique catalysis. Zeolites distribute individual acid sites and/or metallic active sites on regular skeleton structure with high surface area. Owing to the unique environment with molecular-scale pores, periodic structure and highly uniform isolated active centers, zeolites show great potential and excellent performance in industrial applications, such as fluid catalytic cracking (FCC) [1,2], methanol to olefins (MTO) [3,4], dimethyl ether carbonylation to ethanol [5,6] and olefin epoxidation [7,8]. The catalytic behavior of zeolites, governed by both their structural framework and active sites, bears remarkable similarity to metalloenzyme catalysis in biological systems. In metalloenzyme catalytic systems, such as particulate methane monooxygenase (pMMO) for methane selective oxidation to methanol, the magnitude and orientation of electric field from the surrounding microenvironment and the metal active sites determine the catalytic performance, because both of the two counterparts can adjust and optimize the adsorbed position, strength and orientation of the reactant molecules on the active site throughout the catalytic cycle [9−11]. Similar to the metalloenzymes, zeolites with isolated metal atoms embedded into the zeolite framework possess a specific orientation and microenvironment (Fig. 1), which exhibit much better performance than metal clusters and nanoparticles [12−14]. The mutual fusion of metal atom and zeolite generates unique coordination in the framework, resulting in a local metallic center with metal–oxygen (M–O) bonds reinforced by the zeolite microenvironment. So far, there has never been a carrier capable of providing dispersion like zeolites, while reinforcing the active center through the specific topology and coordination environment, and achieving specific orientation with respect to the reactant and intermediate species [15,16].

Alkanes, especially methane and C_2_–C_4_ alkanes, are highly stable molecules, but their abundance and low cost have been the two main driving forces for the development of efficient processes to utilize them. Among the industrial processes, alkane dehydrogenation, such as propane dehydrogenation (PDH), has been employed for alkene production since the 1940s, and two catalyst systems, PtSn/Al_2_O_3_ and CrO*_x_*/Al_2_O_3_, are commercially utilized in Oleflex and CATOFIN technologies, but both suffer from fast deactivation and low propylene selectivity [17,18]. To alleviate this problem, incorporation of metal species into zeolite to develop metal-zeolites (Zn-MFI, Co-MFI etc.) has been demonstrated to be a strategy with great prospects to improve alkane dehydrogenation performance [19−25]. In particular, metal species enter into the zeolite framework by accommodation by zeolite, forming single metal atom sites embedded into the regular skeleton structure and zeolite microenvironment. For decades, metal-zeolites containing oxygen-bridged metal atoms and/or adjacent silanols have been found to exhibit extremely high activity for various reactions, such as alkane activation and biomass conversion [26,27]. However, how the zeolite microenvironment empowers the active center, and how the zeolite environment and metal sites cooperate to carry the reactants to products, and these key scientific issues of metal-zeolite catalysis, are still unanswered despite considerable research efforts in recent years.

Herein, we constructed Cr-MFI zeolite featuring isolated Cr centers, where Cr occupies the T sites of MFI to form a locally distorted tetrahedral structure, comprising one adjacent Si–OH and three Si–O–Cr ({(≡SiO)_3_Cr(≡SiOH)}). We then investigated the catalytic application and mechanism of Cr-MFI in the PDH process and revealed the role of the Cr atom and its surrounding microenvironment. We underscore that a holistic consideration of the combined effects of the metal-zeolite active centers and their microenvironments is crucial for truly understanding the active sites and catalytic mechanisms of metal-zeolites in alkane dehydrogenation. Both the metal center and zeolite microenvironment activate the reactant and participate in the completion of the reaction cycle until the product is generated and leaves the catalyst. Finally, we summarize the Cr-MFI catalysis and propose a Zeo-MOST concept to decipher metal-zeolite catalysis for alkane activation and conversion.

## RESULTS AND DISCUSSION

### Architecture of Cr-MFI

**Figure 2.**
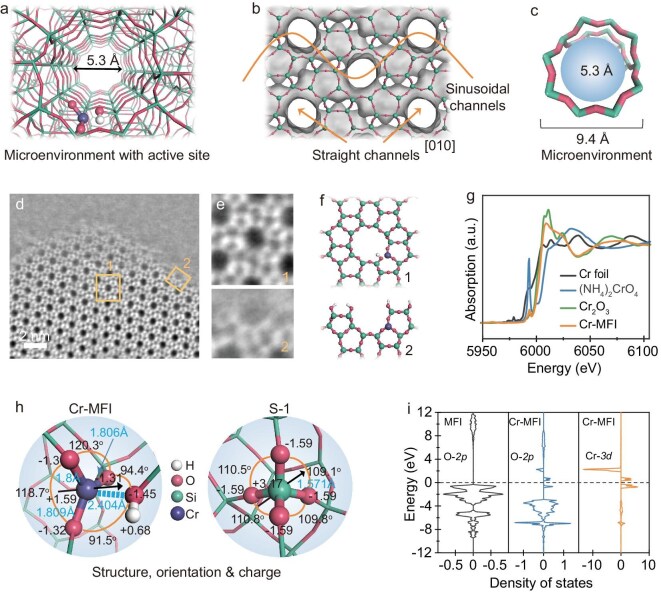
Electronic and coordination structure of Cr-MFI zeolite featuring an atomically dispersed Cr site ({(≡SiO)_3_Cr(≡SiOH)}) in the MFI microenvironment. (a) Overall view of Cr-MFI zeolite: microenvironment embedded with active sites. (b) Straight and sinusoidal channels of Cr-MFI from [010] view. (c) MFI topology with 10-ring channel as the microenvironment. (d) *C_s_*-corrected iDPC-STEM image of Cr-MFI. (e) The enlarged part of the Cr-MFI body and edge in (d). (f) The corresponding models of Cr-MFI in (e). (g) Cr *K*-edge XANES spectra of Cr foil, Cr_2_O_3_, (NH_4_)_2_CrO_4_ and Cr-MFI. The data were collected at the BL14W1 beamline of the Shanghai Synchrotron Radiation Facility (SSRF). (h) Structure, orientation and charge of the Cr-MFI and S-1 at the T_3_ position by DFT calculations. The bond length, O–Cr–O angles, and charges of Si, O and Cr are provided. (i) PDOS of the *p*-orbital of the O atom surrounding the Si atom and the Cr atom, and the *d*-orbital of the Cr atom.

To holistically and accurately present the active centers with their surrounding microenvironment at reaction conditions in metal-zeolites, we constructed a Cr-MFI zeolite with isolated Cr atoms in a purely siliceous MFI zeolite framework (Fig. 2a). X-ray diffraction (XRD) analysis confirms that Cr-MFI exhibits a well-defined MFI structure without detectable CrO*_x_* species (Fig. S1). UV-vis spectra of Cr-MFI zeolites with Cr content below 1.01 wt% display three distinct absorption bands at 276, 357 and 467 nm (Fig. S2), confirming the exclusive presence of isolated Cr–O–Si species. When the Cr loading increases to 1.8 wt%, an additional band emerges at 655 nm, corresponding to the octahedral symmetry T_2g_ ← A_2g_ transition in *α*-Cr_2_O_3_, indicating the formation of Cr_2_O_3_ clusters or nanoparticles [28,29]. Consequently, Cr-MFI with 1.01 wt% Cr was chosen for subsequent investigations. The ^31^P magic angle spinning (MAS) nuclear magnetic resonance (NMR) spectrum of Cr-MFI after trimethylphosphine oxide (TMPO) adsorption-desorption exhibits a single peak at ∼50 ppm (Fig. S3), attributed to TMPO interacting with framework Cr atoms. The absence of correlations in the corresponding 2D spectra further verifies the uniform incorporation of Cr species into the MFI zeolite framework [30–32]. Energy dispersive spectroscopy (EDS) elemental mapping, CD_3_CN-FTIR and pyridine-Fourier transform infrared (FTIR) analyses collectively demonstrate that at 1.01 wt% Cr loading, the Cr species are homogeneously distributed within the MFI framework, exhibiting solely Lewis acidity while maintaining a closed structure (Figs S4–S6, Table S1) [33−35]. The Cr-MFI zeolite possesses a zeolite microenvironment with a well-defined 10-membered ring and straight and sinusoidal channels (Fig. 2b and c). Integrated differential phase contrast scanning transmission electron microscopy (iDPC-STEM) images clearly present the overall architecture of Cr-MFI (Fig. 2d). The presence of complete and partial microenvironments is separately indicated in the bulk and at the edges of MFI zeolite (Fig. 2e and f). To accurately identify the structure and electronic states of Cr-MFI, Cr *K*-edge X-ray absorption spectroscopy (XAS, Fig. 2g) and density functional theory (DFT) analysis are performed, revealing the single Cr center in Cr-MFI, ({(≡SiO)_3_Cr(≡SiOH)}) with Cr–O–Si (3) and Cr···OH–Si (1) (Fig. 2h, Figs S7–S9). This incorporation of Cr atoms into the MFI framework leads to partial loss of the tetrahedral symmetry and gives rise to the distortion of local tetrahedral structures with the radical change in bond length, bond angle and charges of the Cr, O and H atoms, and thereby results in a unique spatial positioning and orientation with distorted tetrahedral microstructure, which are different to those of S-1 zeolite (purely siliceous MFI zeolite). Projected electronic density of states (PDOS) reveals that the O-2*p* orbitals of Cr-MFI overlap the Cr-3*d* orbital owing to the hybridization between Cr-3*d* and O-2*p* states (Fig. 2i), which can facilitate the bonding and coordination of guest reactants through ever-adjusting the electronic states and angles of the Cr centers. Therefore, the MFI skeleton structure and microenvironment accommodating isolated Cr centers with special orientation and tunable electronic state endows special properties to the Cr atom.

### PDH process on Cr-MFI

**Figure 3.**
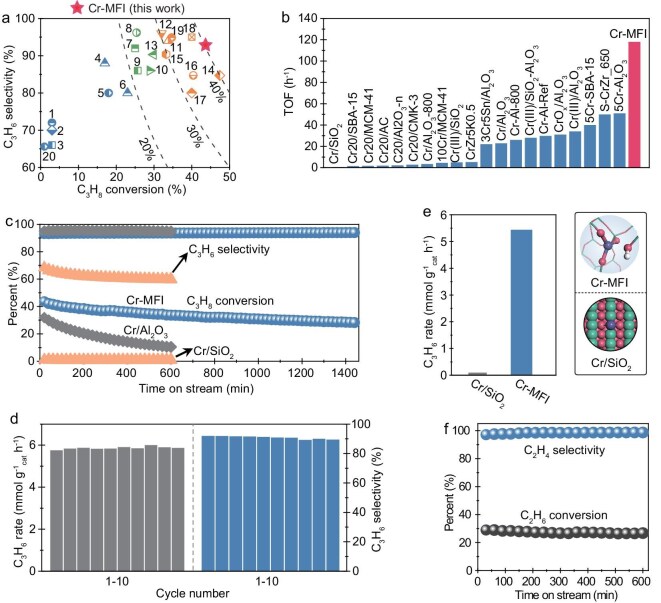
PDH performance of Cr-MFI and Cr-based catalysts. (a) Comparison of the PDH performances over Cr-MFI and previously reported Cr-based catalysts. The detailed comparisons are provided in Table S4. (b) Comparison of the TOF of Cr-based catalysts for PDH. (c) Propane conversion and propylene selectivity over Cr-MFI, Cr/Al_2_O_3_ and Cr/SiO_2_ with time-on-stream. Reaction conditions: 0.2 g, 580°C, weight hourly space velocity (WHSV) = 0.6 h^−1^. (d) C_3_H_6_ formation rate and selectivity over Cr-MFI for 10 cycles. Regeneration conditions: 580°C, air (20 mL min^−1^), 10 min. (e) Initial C_3_H_6_ formation rates of over Cr-MFI and Cr/SiO_2_. (f) Ethane conversion and ethylene selectivity over Cr-MFI with time-on-stream. Reaction conditions: 0.2 g, 5%C_2_H_6_/N_2_, 600°C, WHSV = 0.4 h^−1^.

The obtained Cr-MFI zeolite with Cr active sites orientated in the MFI microenvironment was used in the PDH reaction, which exhibited performance comparable to that of certain Pt-based catalysts and significantly outperformed previously reported transition metal-based systems, underscoring the exceptional PDH activity of Cr-MFI (Fig. 3a and b, Figs S10–S13, Tables S3–S6) [36–46]. Stability tests display that the Cr-MFI possesses very good one-way stability over 24 h time-on-stream and also excellent cycle stability (Fig. 3c and d, Figs S14 and S15). Characterization of the spent Cr-MFI catalyst reveals structural and compositional similarities to the fresh material, confirming its exceptional thermal stability and robust anti-coking resistance (Fig. S16). However, the Cr atoms on amorphous SiO_2_ (Cr/SiO_2_) without zeolite surroundings and a pseudo-tetrahedral structure are almost inactive in the PDH reaction (Fig. 3e, Figs S16–S19), indicating that the Cr-MFI zeolite provides unique catalysis by the zeolite microenvironment and Cr-site orientation specific for PDH to propylene. Furthermore, the Cr-MFI catalyst was used for ethane dehydrogenation, exhibiting good activity and stability (Fig. 3f). It is noteworthy to observe that alkene, being the product of alkane dehydrogenation, generally exhibits high reactivity over acidic zeolites [47,48]. Nevertheless, within the Cr-MFI catalytic environment, subsequent side reactions such as cracking, deep dehydrogenation, polymerization and coking are significantly suppressed. This underscores that the catalytic environment of Cr-MFI not only promotes the primary reaction but also facilitates the elimination of products, thereby preserving the catalyst’s activity and stability. It is a meaningful endeavor to conduct an assessment based on modeling the unique structure of Cr-MFI in order to present its catalytic effects. However, the Cr atom loaded onto amorphous SiO_2_ possesses a complex structure, which is difficult to model.

### Participation of the microenvironment and Cr active center of Cr-MFI in PDH

**Figure 4.**
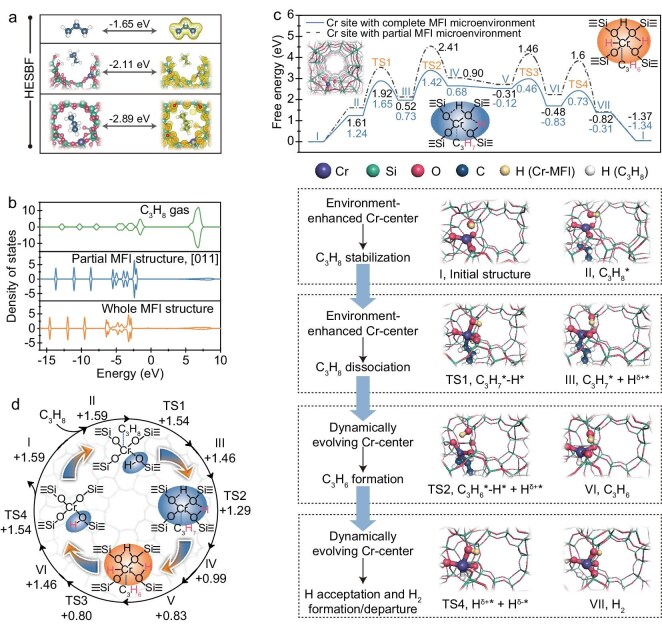
Microenvironment and active site orientation for intensifying propane activation and reaction on Cr-MFI. (a) Charge density of the HESBF for C_3_H_8_ gas in Cr-MFI without, with partial and with whole MFI microenvironment. (b) PDOS analysis of C_3_H_8_ gas in Cr-MFI without, with whole and with partial MFI microenvironment. (c) Free energy diagrams of PDH reaction pathways on Cr-MFI zeolite with whole and partial microenvironment, and the role of active sites with whole microenvironment at different reaction stages. The center, enhanced by the environment, aids in achieving dissociation, and the continuously dynamically evolving Cr center facilitates the formation of propylene, as well as the formation and departure of hydrogen. (d) Dynamic evolution of the Cr electronic states in Cr-MFI with whole microenvironment during PDH reaction by DFT calculations. The numbers are the charges of the Cr atom at the T_3_ site of Cr-MFI during the PDH reaction by DFT calculations.

The role of the Cr active center with a unique microenvironment and orientation in Cr-MFI is clarified by evaluating their participation in PDH with DFT calculations, and Cr-MFI with a Cr atom at the T_3_ site is selected as the representative for the following discussion (Figs S20–S27). The highest electronic states below the Fermi level (HESBF) of C_3_H_8_ molecules shows the energies decrease by 0.46 and 1.24 eV for the C_3_H_8_ molecules in the Cr-MFI with a partial and complete zeolite microenvironment, respectively (Fig. 4a). Furthermore, the density of state of C_3_H_8_ in Cr-MFI shifts downwards as compared with it being placed outside of zeolite (Fig. 4b). These results reveal Cr-MFI zeolite microenvironment accommodates the C_3_H_8_ molecule and offers stabilizing effects during the occurrence of PDH reaction, which facilitates the interplay of Cr-MFI and propane to achieve the conversion.

To make clear the enhanced participations of the Cr active center by the MFI microenvironment, we compared each elementary step during the PDH process on Cr-MFI with complete and partial microenvironments (Fig. 4c, Table S7). For Cr-MFI with a complete microenvironment, the approaching process of the C_3_H_8_ molecule to the Cr center is easier than that in a partial microenvironment, which is proved by the decreased adsorption energies from 1.61 to 1.24 eV. This result is consistent with the above analysis of the decreased HESBF. The adsorbed propane molecule (C_3_H_8_*) will undergo dissociation at the active Cr center {(≡SiO)_3_Cr(≡SiOH)} to form {(≡SiO)_2_Cr(C_3_H_7_) (HOSi≡)_2_} with a reaction free energy of 0.73 eV and a kinetic barrier of 1.65 eV (TS1 in Fig. 4c). Owing to the hybridization between Cr-3*d* and O-2*p* orbitals, the polarized O atoms surrounding the Cr atom can abstract and accept H* from C_3_H_8_* and the C_3_H_7_* moiety stays with the Cr atom. After the first C–H bond dissociation, in the next step, the *β*-H of C_3_H_7_* will dissociate and migrate to the Cr atom, forming Cr–H with a barrier of 1.42 eV (ΔG=0.68 eV, IV in Fig. 4c). Subsequently, C_3_H_6_ desorption on the Cr center produces {(≡SiO)_2_CrH(≡SiOH)_2_} that contains two Cr···OH−Si with hydrogen bonds and one Cr–H bond, which can pool the dissociated H atoms in the Cr center. Noticeably, the H atom in the Cr···OH−Si is positively charged, while the H atom on the Cr atom is negatively charged. Therefore, the H*^δ^*^+^ and H*^δ^*^−^ can be coupled to H_2_ with a low barrier of 0.73 eV (ΔG = −0.31 eV), and H_2_ desorption recovers the original {(≡SiO)_3_Cr(≡SiOH)} active site. In comparison, the whole C_3_H_8_* conversion process in the partial microenvironment is more difficult than that in the complete microenvironment.

Cr-MFI participates in all the steps during the PDH process. The continuous evolution in structure and orientation of the Cr center facilitates the activation of propane, distorting and straining the reactant from its initial state to a transition state, and ultimately leading to the dissociation of the C–H bond and formation of an H–H bond. The coordination and the electronic states of the Cr center (Cr atom and the adjacent O and H atoms) with distorted tetrahedral coordination are constantly evolved in alignment with C–H bond dissociation and H–H bond formation in the MFI zeolite microenvironment (Fig. 4d, Figs S28–S31), which realize highly efficient PDH performance. During the first C–H dissociation, the positive charge of the Cr atom decreases from +1.59 (I and II) to +1.46 (III) and +0.99 (IV). The C_3_H_7_* is then dissociated into C_3_H_6_ and H* on the Cr center with the further charge decrease of the Cr atom from +0.99 (IV) to +0.83 (V). It is notable that a {(≡SiO)CrH(≡SiOH)_2_} with two positively charged H atoms and one negatively charged H atom are formed after the *β*-H dissociation (Fig. 4d). Subsequently, the positive charge of the Cr atom increases from +0.83 (V) to +1.46 (VI) and +1.59 (I) with {(≡SiO)_3_Cr(≡SiOH)} active site restoration to the original state, accompanied by H_2_ formation and departure (Fig. S27). Meanwhile, the electronic states of the adjacent O atoms and the dissociated H* atoms also continuously evolve along the catalytic cycle (Figs S30 and S31), which helps to accelerate the reaction process. The dynamic evolutions of the electronic states of the Cr center correspond to each step of the PDH process, thereby making the PDH process more feasible. As for the Cr-MFI with the Cr center positioning at the external surface with a partial MFI environment, the kinetic barriers (TS1, TS2, TS3 and TS4) for PDH are much higher than those of a Cr site with a complete zeolite microenvironment (Fig. 4c, Fig. S32, Table S8), which confirms the important role of a zeolite microenvironment in Cr-MFI for the alkane dehydrogenation reaction.

The DFT findings demonstrate the exceptional significance of the microenvironment and active site of Cr-MFI in facilitating PDH. Cr-MFI zeolite acts as a catalyst, offering a tailored microenvironment with active sites, which can accommodate and interact with the reaction substrate to achieve its activation and conversion. Throughout the reaction sequence, Cr-MFI dynamically adjusts the orientation and electronic state of its microenvironment and active center to promote the efficient realization of the PDH catalytic cycle.

**Figure 5.**
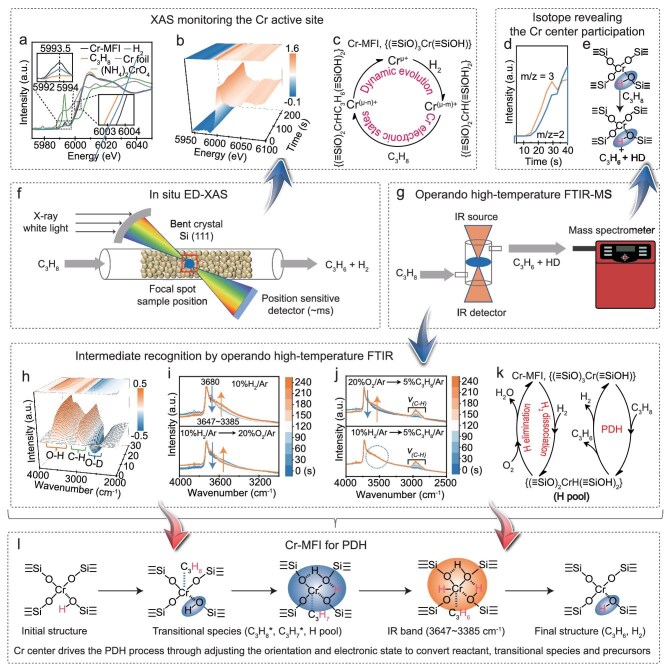
*In situ* XAS and *in situ* high-temperature FTIR spectra identify the dynamic evolution of the electronic states and coordination structure of Cr-MFI during PDH. (a) Cr *K*-edge XANES spectra of the Cr-MFI before and after H_2_ and C_3_H_8_ treatment for 30 min (reaction conditions: 580°C, Ar (10% H_2_/Ar or 5% C_3_H_8_/Ar), 20 mL min^−^^1^). The data were collected at the BL14W1 beamline of the SSRF. (b) *In situ* Cr *K*-edge XANES spectra of the Cr-MFI during PDH. Reaction conditions: 580°C, 5% C_3_H_8_/Ar, 20 mL min^−1^. The data were collected at the BL05U beamline of the SSRF. (c) Schematic illustration of the dynamic evolution of the Cr electronic states in H_2_ and C_3_H_8_ atmosphere at the reaction conditions of (a) and (b). (d) On-line mass spectroscopy analysis of the generated HD (*m*/*z* = 3) and H_2_ (*m*/*z* = 2) in the PDH process over D-labelled Cr-MFI. (e) Schematic illustration of the evolution of D-labelled Cr-MFI for the PDH process. (f) Schematic diagram of the *in situ* time-resolved energy-dispersive XAS (ED-XAS) devices. (g) Schematic diagram of the *in situ* high-temperature FTIR spectroscopy devices with on-line mass spectrometer. (h) *In situ* FTIR spectroscopy study of D-labeled Cr-MFI during PDH. Reaction conditions: 5% C_3_H_8_/Ar, 20 mL min^−1^, 580°C. (i) *In situ* high-temperature FTIR spectra of Cr-MFI in 10% H_2_/Ar (top) and switching transients to 20% O_2_/Ar (down). (j) *In situ* high-temperature FTIR spectra of Cr-MFI in 20% O_2_/Ar and switching transients to 5% C_3_H_8_/Ar (top), 10% H_2_/Ar and switching transients to 5% C_3_H_8_/Ar (down). The reaction conditions in (i) and (j): 580°C and 20 mL min^−1^. (k) Schematic illustration of the evolution of the H network in different atmospheres. (l) Schematic illustration of the isolated Cr center in Cr-MFI for the PDH reaction.

X-ray absorption near-edge structure (XANES) spectra provide evidence for the dynamic progression of the electronic states of the active Cr centers, highlighting the intricate and evolving nature of the electronic configuration of Cr-MFI. The pre-edge peak centered at 5993.5 eV (a combination of quadrupolar 1*s* → 3*d* and dipolar 1*s* → 4*p* transitions) decreases in intensity and the white line shifts to lower energy after H_2_ and C_3_H_8_ pretreatment (Fig. 5a), suggesting the decrease of the positive electricity of the Cr atom in the reaction atmosphere of H_2_ or C_3_H_8_ [49,50]. This verifies the proposal in the previous section (Fig. 4c): the Cr center accepts the propyl species after the dissociation of propane, and varies the electronic state of the Cr-MFI catalyst. Also, *in situ* XANES experiments track the decline of the electronic positivity of the Cr atom in the PDH reaction (Fig. 5b and f). When the Cr-MFI is treated in the flow of 5%H_2_/He at 580°C, the band of Cr *K*-edge XANES spectra at 5993.2 eV decreases until ∼200 s time-on-stream (Fig. S33), then 5% C_3_H_8_/Ar is introduced into the reaction cell by transient switching of the atmosphere, and the white line shifts to lower energy (Fig. 5b), indicating the further decease of the positive electronic states of the Cr atom. Also, the bands at around 6008–6010 eV owing to the electron transfer from ligand to metal atom show a slight increase after introducing 5% C_3_H_8_/Ar, which is due to the C_3_H_7_* ligand attached onto the Cr atom [51,52]. This outcome illustrates the dynamic evolution and continuous adjustment of the electronic state of the Cr-MFI throughout the PDH process (Fig. 5c), which can effectively synchronize with and expedite the progression of the reaction.

Deuterium (D)-labeling experiments are performed over the Cr-MFI, and the D-labelled Cr-MFI is employed in the PDH reaction, which is measured by *in situ* high-temperature FTIR spectroscopy equipped with an on-line mass spectrometer (Fig. 5g, Fig. S34). Upon C_3_H_8_ introduction into the Cr-MFI catalyst, the on-line mass spectrometry detects the appearance of HD followed by the appearance of H_2_ at the initial reaction stage (Fig. 5d and e, Fig. S35). Also, the FTIR bands of the OD groups (2340–2800 cm^−1^) on the zeolite surface progressively decrease in intensity with the recovery of OH groups (3300–3760 cm^−1^, Fig. 5h). All these suggest the direct participation of Cr-MFI for transformation. After the introduction and activation of propane, the catalytic microenvironment of Cr-MFI facilitates the dissociation of propane and positions the dissociated intermediates (C_3_H_7_* and H*) on the Cr center. This aligns with the elementary steps outlined from I to III (depicted in Fig. 4c), where the distinctive Cr-MFI catalyst activates the C–H bond of propane, achieves dissociation, and hosts the intermediates on the Cr center, ultimately resulting in the formation of propylene and hydrogen. The deuterium atoms pre-incorporated into Cr-MFI through deuteration are detected in the product hydrogen, indicating that during the PDH process catalyzed by Cr-MFI (whether in its hydrogenated or deuterated form), the Cr-MFI microenvironment not only activates and dissociates the C–H bonds but also functions as a receptor for hydrogen species intermediates. This facilitates the formation and release of H_2_, thereby enabling the completion of the dehydrogenation process. *In situ* high-temperature Fourier transform infrared (FTIR) spectroscopy clarifies the participation way of Cr-MFI in the PDH process (Fig. 5i and j, Fig. S36). Treating the catalyst in the flow of 10% H_2_/Ar at 580°C, the band at 3680 cm^−1^ (silanol around Cr atom, Cr···OH−Si) decreases progressively in intensity with the rise of the band at 3647–3385 cm^−1^ (hydrogen bonds network) (Fig. 5i). These two bands are recovered in an oxygen atmosphere and exhibit very good reversibility (Fig. S37), suggesting the dynamic and reversible participation of Cr-MFI and quitting from the reaction. When propane is fed onto the catalyst, the interaction between Cr-MFI and propane exhibits a consistent evolution trend (Fig. 5j). This gives strong evidence that the Cr-MFI catalyst not only dissociates the C–H bond to form propylene, but also provides the Cr center to pool the dissociated H species (Fig. 5k). Subsequently, the combination of two H species within the {(≡SiO)CrH(≡SiOH)_2_} complex results in the formation of H–H bonds, ultimately leading to the release of H_2_ gas.

Multiple *in situ* spectroscopic investigations have evidenced the pivotal role of dynamic participation of Cr-MFI during the PDH, echoing the previous catalysis assessment of Cr-MFI throughout the PDH process. Both theoretical simulations and experimental evidence underscore the fact that Cr-MFI, through the concerted interaction of its active centers and surrounding environments, executes all pivotal steps in the propane conversion, including stabilization, capture and subsequent activation, C–H bond dissociation, propylene and hydrogen formation, and finally their departure from the catalyst bed. The proposal and subsequent validation of the mechanism, that the dynamic evolution of the zeolite environment and the orientation of the incorporated metal center enhance the PDH catalytic effect, provide a more profound insight into the catalytic mechanism of metal-containing zeolite catalysts (Fig. 5l).

### Zeo-MOST deciphers metal-zeolite catalysis in alkane dehydrogenation

**Figure 6.**
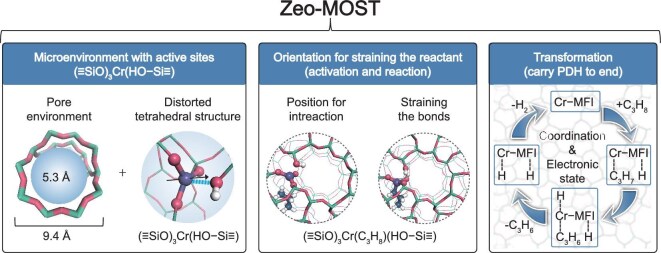
Zeo-MOST deciphers metal-zeolite catalysis for alkane dehydrogenation. Microenvironment (M) with active site Orientation (O) leading to the C–H bond Straining (S) and propane Transformation (T).

Metal-zeolites, exemplified by Cr-MFI, possess a zeolite microenvironment featuring a Cr center ({(≡SiO)_3_Cr(≡SiOH)}) with unique orientation (Fig. 6, left). Upon the guest alkane molecule contacting the Cr-MFI zeolite, its zeolite microenvironment and Cr center provide a special electric field and facilitate reactant activation, thereby enhancing the thermodynamic and kinetic feasibility of alkane dehydrogenation. The active site ({(≡SiO)_3_Cr(≡SiOH)}) then cooperates with the microenvironment and offers an optimal and dynamic orientation and structure for alkane adsorption and activation through straining or compressing the chemical bonds, ultimately enabling dissociation of C–H bonds and formation of H–H bonds (Fig. 6, middle). It should be noticed that the driving force behind alkane dehydrogenation catalyzed by metal-zeolite originates from the Cr center surrounded by the confined electric field and the zeolite microenvironment. This combined catalytic system dynamically evolves and participates in all the alkane dehydrogenation steps from start to end (Fig. 6, right).

Based on the aforementioned results, we herein propose a Zeo-MOST concept to elucidate the mechanism of metal-zeolite catalysis in alkane dehydrogenation (Fig. 6). In the PDH process catalyzed by Cr-MFI, the synergistic interplay between the zeolite microenvironment (M) and the active site orientation (O) achieves the straining (S) of the C–H bonds in propane, ultimately leading to its efficient transformation (T) to the target product. The dynamic evolution of the Cr center in conjunction with the zeolite microenvironment orchestrates every step of propane conversion. The continuous implementation of the Zeo-MOST mechanism enables highly efficient completion of the PDH reaction. This implies that an efficient metal-zeolite catalyst for alkane dehydrogenation performance should possess three critical characteristics: (i) isolated metal active atoms with flexible electronic states and well-defined coordinated structures embedded within a pure silica zeolite framework; (ii) a tailored microenvironment featuring a distinctive electric field distribution that facilitates alkane adsorption, activation and transformation; and (iii) synergistic interplay involving continuous dynamic evolution and cooperative functionality between metal active sites and the zeolite microenvironment throughout the entire catalytic alkane dehydrogenation cycle.

## CONCLUSION

In this study, we have achieved the construction of the metal-incorporated zeolite Cr-MFI, and its application in the PDH reaction. Taking this as an example, combined with theoretical simulations and *in situ* tracking of the catalyst participation by *in situ* XAS, *in situ* FTIR and an isotope labeling technique, we reveal the unique catalysis of enhanced PDH reaction on specifically oriented Cr centers within the zeolite catalytic microenvironment. Cr-MFI has been successfully constructed, where the Cr atom occupies the T sites of MFI to form a local distorted tetrahedral structure comprising one adjacent Si–OH and three Si–O–Cr ({(≡SiO)_3_Cr(≡SiOH)}). The architecture of the Cr-MFI system elucidates that the zeolite framework, in conjunction with its intricate microenvironment, hosts Cr atoms to generate isolated Cr centers possessing precise orientations. This Cr center, along with their adjacent O atoms, exhibits finely tunable electronic configurations. These distinctive structural arrangements of Cr-MFI zeolite impart remarkable and unparalleled properties in the PDH reaction with extremely high activity, selectivity and stability as compared with previous reported Cr-based catalysts. Both theoretical and multiple *in situ* spectroscopic evidence highlights the crucial role of the Cr-MFI microenvironment and active Cr center in all PDH steps. By dynamically adjusting its microenvironment and active center’s orientation and electronic state, Cr-MFI can accommodate and interact with the reaction substrate to achieve its activation and conversion, enabling C–H bond dissociation and H–H bond formation, and promoting efficient PDH catalytic cycles. Cr-MFI, through its active centers and microenvironment, facilitates all key steps in propane conversion, including accommodation, activation, bond dissociation and product formation. These provide deeper insights into the reinforced catalysis from dynamical interplay between the metallic center and its surrounding microenvironment in metal-zeolite materials.

We introduce the Zeo-MOST concept to elucidate the powerful and targeted catalysis of metal-zeolite catalysis in alkane dehydrogenation, especially in the PDH reaction. The zeolite microenvironment (M) and the metallic active site orientation (O) play a pivotal role in achieving the straining (S) of C–H bonds in propane, which ultimately leads to its efficient transformation (T) into the target product. The dynamic evolution of the Cr center, in conjunction with the zeolite microenvironment, orchestrates and fine-tunes each step of the propane conversion process. Continuously implementing the Zeo-MOST mechanism achieves highly efficient completion of the PDH reaction from its inception to its conclusion. This concept not only provides a deeper understanding of metal-zeolite catalysis but also offers valuable insights for the rational design and optimization of such catalysts for industrial applications.

## Supplementary Material

nwaf405_Supplemental_File

## References

[1] Primo A, Garcia H. Zeolites as catalysts in oil refining. Chem Soc Rev 2014; 43: 7548–61. 10.1039/C3CS60394F 24671148

[2] Vogt ETC, Weckhuysen BM. Fluid catalytic cracking: recent developments on the grand old lady of zeolite catalysis. Chem Soc Rev 2015; 44: 7342–70. 10.1039/C5CS00376H 26382875 PMC4594121

[3] Ye M, Tian P, Liu Z. DMTO: a sustainable methanol-to-olefins technology. Engineering 2021; 7: 17–21. 10.1016/j.eng.2020.12.001

[4] Tian P, Wei Y, Ye M et al. Methanol to olefins (MTO): from fundamentals to commercialization. ACS Catal 2015; 5: 1922–38. 10.1021/acscatal.5b00007

[5] Chemical Engineering . Debut of a Coal-To-Ethanol Plant. https://www.chemengonline.com/debut-coal-ethanol-plant/ (28 September 2025, date last accessed).

[6] Cao K, Fan D, Li L et al. Insights into the pyridine-modified MOR zeolite catalysts for DME carbonylation. ACS Catal 2020; 10: 3372–80. 10.1021/acscatal.9b04890

[7] Gordon CP, Engler H, Tragl AS et al. Efficient epoxidation over dinuclear sites in titanium silicalite-1. Nature 2020; 586: 709–12. 10.1038/s41586-020-2826-3 33116285

[8] Grosso-Giordano NA, Hoffman AS, Boubnov A et al. Dynamic reorganization and confinement of Ti^IV^ active sites controls olefin epoxidation catalysis on two-dimensional zeotypes. J Am Chem Soc 2019; 141: 7090–106. 10.1021/jacs.9b02160 30955340

[9] Ross MO, MacMillan F, Wang J et al. Particulate methane monooxygenase contains only mononuclear copper centers. Science 2019; 364: 566–70. 10.1126/science.aav2572 31073062 PMC6664434

[10] Lieberman RL, Rosenzweig AC. Crystal structure of a membrane-bound metalloenzyme that catalyses the biological oxidation of methane. Nature 2005; 434: 177–82. 10.1038/nature03311 15674245

[11] Banerjee R, Lipscomb JD. Small-molecule tunnels in metalloenzymes viewed as extensions of the active site. Acc Chem Res 2021; 54: 2185–95. 10.1021/acs.accounts.1c00058 33886257 PMC8130187

[12] Snyder BER, Bols ML, Rhoda HM et al. Cage effects control the mechanism of methane hydroxylation in zeolites. Science 2021; 373: 327–31. 10.1126/science.abd5803 34437151 PMC10353845

[13] Sushkevich VL, Palagin D, Ranocchiari M et al. Selective anaerobic oxidation of methane enables direct synthesis of methanol. Science 2017; 356: 523–7. 10.1126/science.aam9035 28473586

[14] Chai Y, Han X, Li W et al. Control of zeolite pore interior for chemoselective alkyne/olefin separations. Science 2020; 368: 1002–6. 10.1126/science.aay8447 32467390

[15] Snyder BER, Bols ML, Schoonheydt RA et al. Iron and copper active sites in zeolites and their correlation to metalloenzymes. Chem Rev 2018; 118: 2718–68. 10.1021/acs.chemrev.7b00344 29256242

[16] Liu L, Corma A. Isolated metal atoms and clusters for alkane activation: translating knowledge from enzymatic and homogeneous to heterogeneous systems. Chem 2021; 7: 2347–84. 10.1016/j.chempr.2021.04.001

[17] Sattler JJHB, Ruiz-Martinez J, Santillan-Jimenez E et al. Catalytic dehydrogenation of light alkanes on metals and metal oxides. Chem Rev 2014; 114: 10613−53. 10.1021/cr5002436 25163050

[18] Hannagan RT, Giannakakis G, Réocreux R et al. First-principles design of a single–atom–alloy propane dehydrogenation catalyst. Science 2021; 372: 1444–7. 10.1126/science.abg8389

[19] Zeng L, Cheng K, Sun F et al. Stable anchoring of single rhodium atoms by indium in zeolite alkane dehydrogenation catalysts. Science 2024; 383: 998–1004. 10.1126/science.adk5195 38422151

[20] Zhao D, Tian X, Doronkin DE et al. In situ formation of ZnO*_x_* species for efficient propane dehydrogenation. Nature 2021; 599: 234–8. 10.1038/s41586-021-03923-3 34759363 PMC8580824

[21] Zhou H, Yi X, Hui Y et al. Isolated boron in zeolite for oxidative dehydrogenation of propane. Science 2021; 372: 76–80. 10.1126/science.abe7935 33795454

[22] Ryoo R, Kim J, Jo C et al. Rare-earth–platinum alloy nanoparticles in mesoporous zeolite for catalysis. Nature 2020; 585: 221–4. 10.1038/s41586-020-2671-4 32908262

[23] Hu ZP, Qin G, Han J et al. Atomic insight into the local structure and microenvironment of isolated Co-motifs in MFI zeolite frameworks for propane dehydrogenation. J Am Chem Soc 2022; 144: 12127−37. 10.1021/jacs.2c02636 35762495

[24] Xu Z, Gao M, Wei Y et al. Pt migration–lockup in zeolite for stable propane dehydrogenation catalyst. Nature 2025; 643; 691–8. 10.1038/s41586-025-09168-8 40436038

[25] Hong H, Xu Z, Mei B et al. A self-regenerating Pt/Ge-MFI zeolite for propane dehydrogenation with high endurance. Science 2025; 383; 497–502. 10.1126/science.adu6907 40208961

[26] Ferrini P, Dijkmans J, Clercq RD et al. Lewis acid catalysis on single site Sn centers incorporated into silica hosts. Coordin Chem Rev 2017; 343: 220–55. 10.1016/j.ccr.2017.05.010

[27] Suib SL, Přech J, Szaniawska E et al. Recent advances in tetra- (Ti, Sn, Zr, Hf) and pentavalent (Nb, V, Ta) metal-substituted molecular sieve catalysis. Chem Rev 2023; 123: 877–917. 10.1021/acs.chemrev.2c00509 36547404

[28] Weckhuysen BM, Wachs IE, Schoonheydt RA. Surface chemistry and spectroscopy of chromium in inorganic oxides. Chem Rev 1996; 96: 3327–49. 10.1021/cr940044o 11848862

[29] Gao J, Zheng Y, Tang Y et al. Spectroscopic and computational study of Cr oxide structures and their anchoring sites on ZSM‑5 zeolites. ACS Catal 2015; 5: 3078–92. 10.1021/acscatal.5b00333

[30] Zheng A, Liu SB, Deng F. ^31^P NMR chemical shifts of phosphorus probes as reliable and practical acidity scales for solid and liquid catalysts. Chem Rev 2017; 117: 12475−531. 10.1021/acs.chemrev.7b00289 28952317

[31] Dubray F, Moldovan S, Kouvatas C et al. Direct evidence for single molybdenum atoms incorporated in the framework of MFI zeolite nanocrystals. J Am Chem Soc 2019; 141: 8689–93. 10.1021/jacs.9b02589 31117550

[32] Yi X, Chen W, Xiao Y et al. Spectroscopically visualizing the evolution of hydrogen-bonding interactions. J Am Chem Soc 2023; 145: 27471−9. 10.1021/jacs.3c08723 37993784

[33] Abidi N, Boudjema Y, Rivallan M et al. Challenging the distinction between “open” and “closed” Sn sites in β zeolite by deuterated acetonitrile adsorption: experimental and theoretical insights. J Phys Chem C 2025; 129: 14011−9. 10.1021/acs.jpcc.5c03670

[34] Alghannam A, Bell AT. Effects of cofeeding hydrogen on propane dehydrogenation catalyzed by isolated iron sites incorporated into dealuminated BEA. J Am Chem Soc 2025; 147: 1677–93. 10.1021/jacs.4c12344 39746209

[35] Yuan Y, Brady C, Annamalai L et al. Ga speciation in Ga/H-ZSM-5 by in-situ transmission FTIR spectroscopy. J Catal 2021; 393: 60–9. 10.1016/j.jcat.2020.11.004

[36] Delley MF, Silaghi MC, Nuñez-Zarur F et al. X−H bond activation on Cr(III),O sites (X = R, H): key steps in dehydrogenation and hydrogenation processes. Organometallics 2017; 36: 234–44. 10.1021/acs.organomet.6b00744

[37] Kumar MS, Hammer N, Rønning M et al. The nature of active chromium species in Cr-catalysts for dehydrogenation of propane: new insights by a comprehensive spectroscopic study. J Catal 2009; 261: 116–28.

[38] Han S, Otroshchenko T, Zhao D et al. The effect of ZrO_2_ crystallinity in CrZrO*_x_*/SiO_2_ on non-oxidative propane dehydrogenation. Appl Catal A 2020; 590: 117350. 10.1016/j.apcata.2019.117350

[39] Hu ZP, Wang Z, Yuan ZY. Cr/Al_2_O_3_ catalysts with strong metal-support interactions for stable catalytic dehydrogenation of propane to propylene. Mol Catal 2020; 493: 111052.

[40] Węgrzyniak A, Jarczewski S, Węgrzynowicz A et al. Catalytic behavior of chromium oxide supported on nanocasting-prepared mesoporous alumina in dehydrogenation of propane. Nanomaterials 2017; 7: 249. 10.3390/nano7090249 28862670 PMC5618360

[41] Węgrzyniak A, Rokicińska A, Hędrzak E et al. High-performance Cr–Zr–O and Cr–Zr–K–O catalysts prepared by nanocasting for dehydrogenation of propane to propene. Catal Sci Technol 2017; 7: 6059–68. 10.1039/C7CY01744H

[42] Węgrzyniak A, Jarczewski S, Wach A et al. Catalytic behaviour of chromium oxide supported on CMK-3 carbon replica in the dehydrogenation propane to propene. Appl Catal A 2015; 508: 1–9. 10.1016/j.apcata.2015.10.002

[43] Gao XQ, Lu WD, Hu SZ et al. Rod-shaped porous alumina-supported Cr_2_O_3_ catalyst with low acidity for propane dehydrogenation. Chin J Catal 2019; 40: 184–91. 10.1016/S1872-2067(18)63202-4

[44] He D, Zhang Y, Yang S et al. Investigation of the isolated Cr(VI) species in Cr/MCM-41 catalysts and its effect on catalytic activity for dehydrogenation of propane. ChemCatChem 2018; 10: 5434–40. 10.1002/cctc.201801598

[45] Han S, Zhao Y, Otroshchenko T et al. Unraveling the origins of the synergy effect between ZrO_2_ and CrO_x_ in supported CrZrO_x_ for propene formation in nonoxidative propane dehydrogenation. ACS Catal 2020; 10: 1575–90. 10.1021/acscatal.9b05063

[46] Zhang L, Chen K, Chen H et al. Elucidating the promoting advantages and fundamentals for their creation in Sn-modified commercial CrO_x_/Al_2_O_3_ catalyst for propane dehydrogenation. Chem Eng J 2024; 483: 149366. 10.1016/j.cej.2024.149366

[47] Song Y, Zhu X, Xie S et al. The effect of acidity on olefin aromatization over potassium modified ZSM-5 catalysts. Catal Lett 2004; 97: 31–6. 10.1023/B:CATL.0000034281.58853.76

[48] Cnudde P, Wispelaere KD, Vanduyfhuys L et al. How chain length and branching influence the alkene cracking reactivity on H‑ZSM‑5. ACS Catal 2018; 8: 9579–95. 10.1021/acscatal.8b01779 30319885 PMC6179455

[49] Trummer D, Searles K, Algasov A et al. Deciphering the Phillips catalyst by orbital analysis and supervised machine learning from Cr pre-edge XANES of molecular libraries. J Am Chem Soc 2021; 143: 7326–41. 10.1021/jacs.0c10791 33974429

[50] Barzan C, Piovano A, Braglia L et al. Ligands make the difference! Molecular insights into Cr^VI^/SiO_2_ Phillips catalyst during ethylene polymerization. J Am Chem Soc 2017; 139: 17064−73. 10.1021/jacs.7b07437 28826217

[51] Demmelmaier CA, White RE, Bokhoven JA et al. Evidence for a chromasiloxane ring size effect in Phillips (Cr/SiO_2_) polymerization catalysts. J Catal 2009; 262: 44–56. 10.1016/j.jcat.2008.11.024

[52] Groppo E, Prestipino C, Cesano F et al. In situ, Cr *K*-edge XAS study on the Phillips catalyst: activation and ethylene polymerization. J Catal 2005; 230: 98−108. 10.1016/j.jcat.2004.11.017

