# Supplementary material for: Synergy principle of single active centers and microenvironment for Cr-MFI-catalyzed alkane dehydrogenation
Source: Natl Sci Rev. 2025 Sep 22;12(11):nwaf405. doi: 10.1093/nsr/nwaf405 (PMC12621223; doi:10.1093/nsr/nwaf405)
Supplement: nwaf405_Supplemental_File [file nwaf405_supplemental_file.pdf]

Supplementary materials

Synergy principle of single active centers and microenvironment for Cr-MFI catalyzed alkane dehydrogenation

Zhong-Pan Hu[1,†], Gangqiang Qin[2,7,†], Jingfeng Han[1], Yijun Zheng[1], Zhen Liu[3], Yong Jiang[3], Xiaozhi Su[3], Te Ji[3], Min Li[4], Zhong-Yong Yuan[5], Jianping Xiao[2,7,]*, Svetlana Mintova[6], Yingxu Wei[1,]* and Zhongmin Liu[1,2,7,8,]*

[1]National Engineering Research Center of Lower-Carbon Catalysis Technology, Dalian National Laboratory for Clean Energy, Collaborative Innovation Center of Chemistry for Energy Materials (iChEM), Dalian Institute of Chemical Physics, Chinese Academy of Sciences, Dalian 116023, China;

[2]State Key Laboratory of Catalysis, Dalian Institute of Chemical Physics, Chinese Academy of Sciences, Dalian 116023, China;

[3] Shanghai Synchrotron Radiation Facility (SSRF), Shanghai Institute of Applied Physics, Chinese Academy of Sciences, Shanghai 201800, China;

[4]Elettra–Sincrotrone Trieste, Trieste 34149, Italy;

[5]School of Materials Science and Engineering, Nankai University, Tianjin 300350, China;

[6]Normandie University, Laboratory of Catalysis and Spectrochemistry (LCS), ENSICAEN, UNICAEN, CNRS, Caen 14050, France;

[7]University of Chinese Academy of Sciences, Beijing 100049, China

[8]Lead contact

***Corresponding authors**. E-mails: liuzm@dicp.ac.cn; weiyx@dicp.ac.cn; xiao@dicp.ac.cn

[†]Equally contributed to this work.

Table of Contents

1. Experiments 4

1.1 Materials and catalyst preparation 4

1.2 Catalyst characterizations........ 4

1.3 Computational details 7

1.4 Evaluation of catalytic performance 8

2. Supplementary Figures 10

Figure S1. XRD patterns of S-1 and Cr-MFI with different Cr contents. 10

Figure S2. UV-vis spectra of S-1 and Cr-MFI with different Cr contents. 11

Figure S3. Solid-sate NMR spectra of Cr-MFI. 12

Figure S4. TEM images of Cr-MFI. 13

Figure S5. FTIR spectra of Cr-MFI after CD_3CN adsorption and desorption at different temperatures (323, 373, 473, and 573 K). 14

Figure S6. Pyridine-FTIR of Cr-MFI. 15

Figure S7. FT k^2-weighted EXAFS spectra of Cr foil, Cr_2O_3, $(NH_4)_2CrO_4$, and Cr-MFI. 16

Figure S8. Cr K-edge EXAFS (circle) and fitting curve (line) for Cr-MFI. 16

Figure S9. Local structure of Cr-MFI with Cr at T_1, T_7, T_9, and T_{10} sites by DFT simulation. 17

Figure S11. TPSR results of different Cr-based catalysts. 19

Figure S12. C_3H_6-TPD analysis of Cr-based catalysts. 20

Figure S13. Catalytic results of Cr-MFI, Cr/Al_2O_3, and Cr/HZSM-5. 21

Figure S14. Propane conversion and propylene selectivity of Cr-MFI. 22

Figure S15. Cycle stability of the Cr-MFI. 23

Figure S17. UV-vis spectra of Cr/SiO_2 with Cr content of 1 wt%. 25

Figure S18. XRD patterns of Cr/SiO_2. 25

Figure S19. Propane conversion and propylene selectivity of Cr/SiO_2. 26

Figure S20. Local structure of Cr-MFI with Cr at T_3 site with complete pore environment by DFT simulation.27

Figure S21. DFT calculations of the first C−H bond dissociation of C_3H_8 on Cr-MFI with Cr atom locating at the T_3 site with complete microenvironment 28

Figure S22. Local structure of Cr-MFI with Cr at T_3 site without microenvironment by DFT simulation........ 29

Figure S23. DFT calculations of the first C−H bond dissociation of C_3H_8 on Cr-MFI with Cr atom locating at the T_3 site without complete microenvironment. 30

Figure S24. DFT calculations of the first C−H bond dissociation of C_3H_8 on Cr-MFI with Cr atom locating at the T_1 site with complete microenvironment. 31

Figure S25. DFT calculations of the first C─H bond dissociation of C_3H_8 on Cr-MFI with Cr atom locating at the T_7 site with complete microenvironment. 32

Figure S26. DFT calculations of the first C─H bond dissociation of C_3H_8 on Cr-MFI with Cr atom locating at the T_9 site with complete microenvironment. 33

Figure S27. DFT calculations of the first C─H bond dissociation of C_3H_8 on Cr-MFI with Cr atom locating at the T_{10} site with complete microenvironment. 34

Figure S28. The charges of H, C, O, and Cr atom at T_3 site of Cr-MFI during PDH reaction by DFT calculations........ 35

Figure S29. Dynamic evolution of the Cr electronic states during PDH reaction by DFT calculations. 36

Figure S30. Dynamic evolution of the O electronic states during PDH reaction by DFT calculations. 36

Figure S31. Dynamic evolution of the H electronic states during PDH reaction by DFT calculations. 37

Figure S32. The corresponding structures at different steps over the Cr-MFI zeolite with partial microenvironment. 37

Figure S33. In situ XAS spectra of Cr-MFI pretreated in 10%H_2/He atmosphere at 580 ℃ with time on stream.38

Figure S34. *In situ* FTIR spectroscopy study of Cr-MFI and S-1 pretreated in D_2 atmosphere. 39

Figure S35. On-line mass spectroscopy (MS) analysis of the generated HD (m/z = 3) and H_2 (m/z = 4) in PDH process over D-labelled Cr-MFI. 40

Figure S36. Operando high-temperature FTIR instrument. 40

Figure S37. Operando high-temperature FTIR spectra of Cr-MFI at different atmospheres for another four cycles........ 41

3. Tables 42

Table S1. The physicochemical properties of S-1 and Cr-MFI samples........ 42

Table S2. Structural parameters of Cr-MFI extracted from quantitative EXAFS curve-fitting........ 42

Table S3. Catalytic performance of Cr/SiO_2, Cr/Al_2O_3 and Cr-MFI for PDH at 580 ℃ under different flow conditions. 43

Table S4. Comparison of the Cr-based catalysts for PDH. 44

Table S5. Comparison of the Pt-based catalysts for PDH........ 45

Table S6. Comparison of the transition metal-based catalysts in PDH........ 46

Table S7. The distances of Cr─C, Cr···O, Cr─O, and Cr─H during PDH process on Cr-MFI zeolite with Cr at T_3 site with whole microenvironment. 47

Table S8. The distances of Cr─C, Cr···O, Cr─O, and Cr─H during PDH process on Cr-MFI zeolite with Cr at T_3 site without whole microenvironment. 48

4. References 49

1. Experiments

1.1 Materials and catalyst preparation

Tetraethylorthosilicate (TEOs, 98%), isopropanol (IPA, 99%), and $Cr(NO_3)_3{\cdot}9H_2O$ were purchased from Tianjin Guangfu Fine Chemical Research Institute. Tetrapropylammonium hydroxide solution (TPAOH, 25%) was obtained from Beijing Enochai Technology Co., Ltd. All the chemicals were obtained as received without further purification. SiO_2, Al_2O_3 and HZSM-5 (Si/Al = 28) were provided by Tianjin Shenneng SciTech Co., Ltd.

Chromium (Cr) species incorporated into MFI zeolite were prepared by an in situ hydrothermal method. Typically, 20 g tetraethyl orthosilicate (TEOs) and calculated $Cr(NO_3)_3{\cdot}9H_2O$ were dissolved into 23.4 g tetrapropylammonium hydroxide solution and stirred in an ice-bath for 2 h. Then, the mixture was heated to 70 □ for 4 h and subsequently added with 24 g isopropanol. After stirring for another 2 h, the mixture was transformed into a Teflon-lined autoclave at 170 □ for 72 h. After that, the obtained sample was washed by deionized water and ethanol for several times, dried at 100 □ for 24 h, and followed by calcination at 550 □ for 6 h (heating rate, 2 □ min^{-1}). The contents of Cr were characterized by X-ray fluorescence (XRF) technique.

Silicate-1 (S-1) was prepared by the similar method to Cr-MFI without adding $Cr(NO_3)_3{\cdot}9H_2O$.

Cr/SiO_2, Cr/Al_2O_3, and Cr/HZSM-5 were prepared by a wet impregnation method. Typically, the calculated $Cr(NO_3)_3{\cdot}9H_2O$ was dissolved into 20 mL deionized water. Then, 2 g support (SiO_2, Al_2O_3 or HZSM-5) was added into the above solution, stirred for 4 h, and evaporated at 80 □. After that, the sample was dried at 100 □ for 12 h and calcined at 550 □ for 6 h with a heating rate of 2 □ min^{-1}.

1.2 Catalyst characterizations

X-ray diffraction (XRD) patterns were recorded on a PANalytical X'Pert PRO X-ray diffractometer with Cu Kα radiation (λ = 1.5418 Å). C_s-corrected high-angle annular dark-field-scanning transmission electron microscopy (HAADF-STEM) and integrated differential phase contrast (iDPC) images were tested on FEI Titan Cubed Themis ETEM G3 300. STEM-energy dispersive spectroscopy (STEM-EDS) elemental mapping images were collected by a Jeol JEM-2800 microscope at 200 kV. X-ray fluorescence (XRF) analysis was conducted on a Philips Magix-601 X-ray fluorescence spectrometer. Ultraviolet-visible (UV-*vis*) spectroscopy experiments were performed on a VARIAN Cary-5000 UV-vis-NIR spectrophotometer equipped with an integration sphere in the wavelength range of 200~800 nm. X-ray absorption fine spectroscopy (XAFS) measurements were performed at the BL14W1 beamline of the Shanghai Synchrotron Radiation Facility (SSRF). The EXAFS analysis was performed using the GNXAS package, which is based on multiple scattering theory. Thermogravimetry analysis (TGA) was performed on a TA SDT Q600 instrument. Typically, all the samples were pretreated at 200 ^oC for 1 h. Then, the pretreated sample was heated to 700 ^oC in a flow of air (100 ml min^{-1}) with a heating rate of 10 ^oC

min^{-1}. Raman experiments were performed on a home-made single stage UV Raman spectrograph. The single-frequency UV laser line at 244 nm was from an efficient external cavity frequency doubler (Wavetrain, Spectra-Physics) of the single-frequency laser at 488 nm laser (Genesis, CX 488, Coherent).

N_2 adsorption-desorption isotherms were measured by a Quantachrome Autosorb-1MP sorption analyzer. Prior the measurement, all the samples were degassed at 350 ℃ for 3 h. Then, the N_2 sorption tests were conducted at −196 ℃. The pore volume was obtained at P/P_0 = 0.97. The specific surface area was calculated by the Brunauer-Emmett-Teller (BET) method.

Propane temperature-programed surface reaction (C_3H_8-TPSR) tests were carried out in a fixed-bed micro-reactor equipped with an on-line mass spectrometer (Shimadzu MS-QP2010 SE). Typically, 0.2 g sample was pre-reduced in pure H_2 flowing (20 mL min^{-1}) at 580 ℃ for 1 h and then purged with Ar flowing for 0.5 h. After cooling down to room temperature, 5%C_3H_8/Ar (20 mL min^{-1}) was passed through the pretreated catalysts. Until the baseline flat, the sample was heated from room temperature to 650 ℃ with a heating rate of 10 ℃ min^{-1}. The products, including hydrogen (m/z = 2), methane (m/z = 16), ethylene (m/z = 26), ethane (m/z = 30), propylene (m/z = 42) and propane (m/z = 43), are detected by the on-line mass spectrometer.

Propylene temperature-programmed desorption (C_3H_6-TPD) experiments were conducted in a fix-bed micro-reactor equipped with a mass spectrometer (Shimadzu MS-QP2010 SE). Before the tests, 0.2 g sample was pre-reduced in H_2 flowing (20 mL min^{-1}) at 580 ℃ for 30 min and then purged by Ar flowing for about 0.5 h. After cooling down to 100 ℃, the degassed sample was exposed in a flow of 5%C_3H_6/Ar (20 mL min^{-1}) for 30 min, and then purged by a flow of Ar (20 mL min^{-1}). Until the baseline flat, the resultant sample was heated to the desired temperature by a heating rate of 10 ℃ min^{-1}. The desorbed components were measured by an on-line mass spectrometer.

Fourier transform infrared (FTIR) spectroscopy experiments of CD_3CN adsorption and desorption were performed on a Bruker Vextex 70 spectroscope equipped with a mercury-cadmium-telluride (MCT) detector. First, ~25 mg sample was tableted into a disk (r = 7 mm). Then, the small rounds were put into the heating pool and heated to 500 ℃ for 1 h. After cooling down to room temperature, the desorbed sample was saturated with pyridine and subsequently heated to 50, 100, 200, and 300 ℃ for 0.5 h. All the spectra were measured at the desired temperature with 32 scans at a resolution of 4 cm^{-1}.

FTIR experiments of pyridine adsorption and desorption were carried out on a Brukers Optics XF808-04 spectrometer. First, ~25 mg sample was tableted into a disk (r = 7 mm). Then, the small rounds were put into the heating pool and heated to 500 ℃ for 1 h. After cooling down to room temperature, the desorbed sample was saturated with pyridine and subsequently heated to 200, 300, and 350 ℃ for 0.5 h. All the spectra were measured

at the desired temperature with 32 scans at a resolution of 4 cm^{-1}. The acid density of the sample was calculated as follows [1]:

$$C_B = 1.88 \times I_B \times R^2/W \tag{7}$$

$$C_L = 1.42 \times I_L \times R^2/W \tag{8}$$

C_B (mmol g_{cat}^{-1}) and C_L (mmol g_{cat}^{-1}) represent the concentration of Brønsted and Lewis acid sites, respectively. I_B and I_L are the integrated absorbance of bands at 1,540 and 1,450 cm^{-1}, respectively. R (cm) and W (mg) are the radius and weight of the disk, respectively.

FTIR experiments about C_3H_6 adsorption and desorption were performed on a Bruker Vextex 70 spectroscope equipped with a mercury-cadmium-telluride (MCT) detector. Firstly, the sample was pretreated in H_2 flowing (20 mL min^{-1}) at 580 ℃ for 30 min. Then, the sample was cooled down to room temperature and purged by He (20 mL min^{-1}). After the spectra were stable, the background was collected. Then, 5%C_3H_6/He was introduced into the reactor, and the FTIR spectra were recorded. About 30 min later, the sample was purged by He flowing (20 mL min^{-1}).

Solid-state MAS nuclear magnetic resonance (NMR) tests were carried out on a Bruker Avanve III 600 spectrometer at 12 kHz, using zirconia rotors (*i.d.* = 4 mm) with a spinning frequency of 12 kHz. For ^{29}Si MAS NMR, a single pulse excitation (30° flip angle) is used with a recycle delay of 30 s. For cross-polarization (CP) {^{1}H} ^{29}Si MAS NMR, the contact time and recycle delay are 5 ms and 2 s, respectively. The ^{31}P NMR spectra of Cr-MFI were characterized by monitoring the adsorption of trimethylphosphine oxide (TMPO) by ^{31}P-NMR under ^{1}H decoupling. All the preparation steps were conducted under Ar atmosphere. Before the test, the Cr-MFI was dehydrated at 400 ℃ under vacuum for 12 h. The TMPO was dissolved in dichloromethane. Then, the TMPO solution was added into the dehydrated Cr-MFI and maintained for 1 h. Subsequently, the sample was evaporated under vacuum at room temperature or 165 ℃ for 2 h. The resulting sample was put into zirconium rotors for ^{31}P MAS NMR.

The in situ XAS experiments were performed at the SSRF (BL05U) beamline. A double-crystal Si (111) monochromator with continuous scanning in transmission mode was used to collect XAS spectra at the Cr K-edge. Typically, 100 mg sample was tableted in a round (r = 4 mm) and put it into an in situ cell at 580 ℃ in Ar (20 mL min^{-1}) for 30 min. Then, 10%H_2/He (20 mL min^{-1}) was introduced into the reactor for 30 min. After purifying by He, 5%C_3H_8/He (20 mL min^{-1}) was introduced into the reactor. Meanwhile, all the XAS spectra were collected.

All the in situ high-temperature FTIR experiments were performed on a Bruker Vextex 70v spectroscope equipped with a MCT detector. 10%H_2/Ar experiments: Typically, 10 mg sample was tableted into a round (r = 7 mm) and put it into an in situ cell at 580 ℃ in Ar (20 mL min^{-1}) for 30 min. Then, 10%H_2/Ar (20 mL min^{-1}) was introduced into the reactor for 30 min. 10%H_2/Ar → 20%O_2/Ar experiments: After the 10%H_2/Ar treatment, Ar (20 mL min^{-1}) was shifted into the reactor for 10 min. Then, 20%O_2/Ar (20 mL min^{-1}) was introduced into the reactor. 20%O_2/Ar → 5%C_3H_8/Ar experiments: Typically, 10 mg sample was tableted into a round (r = 7 mm) and put it into an in situ cell at 580 ℃ in Ar (20 mL min^{-1}) for 30 min. Then, 20%O_2/Ar (20 mL min^{-1}) was introduced into the reactor for 30 min. After purified by Ar (20 mL min^{-1}), 5%C_3H_8/Ar (20 mL min^{-1}) was shifted into the reactor. 10%H_2/Ar → 5%C_3H_8/Ar experiments: Typically, 10 mg sample was tableted into a round (r = 7 mm) and put it into an in situ cell at 580 ℃ in Ar (20 mL min^{-1}) for 30 min. Then, 10%H_2/Ar (20 mL min^{-1}) was introduced into the reactor for 30 min. After purified by Ar (20 ml min^{-1}), 20%O_2/Ar (20 mL min^{-1}) was introduced into the reactor.

All the deuterium related FTIR experiments were performed on a Bruker Vextex 70v spectroscope equipped with a MCT detector. H-D exchange experiments: Typically, 10 mg sample was tableted into a round (r = 7 mm) and put it into an in situ cell at 580 ℃ in Ar (20 mL min^{-1}) for 30 min. Then, 5%D_2/Ar (20 mL min^{-1}) was introduced into the reactor. D-labelled Cr-MFI for PDH: Typically, the Cr-MFI was treated by 5%D_2/Ar (20 mL min^{-1}) at 580 ℃ for 1 h. Then, 5%C_3H_8/Ar (20 mL min^{-1}) was shifted into the reactor. PDH reaction of C_3D_8: Typically, 10 mg sample was tableted into a round (r = 7 mm) and put it into the in situ cell of FTIR at 580 ℃ in Ar (20 mL min^{-1}) for 30 min. Then, 5%C_3D_8/Ar was shifted into the reactor.

1.3 Computational details

All the calculations based on spin-polarized density functional theory (DFT) were performed by using Vienna ab initio simulation package (VASP 5.3.5) [2−11]. The generalized gradient approximation (GGA) and RPBE functional was applied [2,8]. The interactions between valence electrons are described by PAW [6], and the cutoff energy is set to be 400 eV. The geometry optimizations were converged when the maximal force is smaller than 0.05 eV/Å, and the convergence threshold of energy was set as 10^{-5} eV. The climbing image nudged elastic band (CINEB) method is used to searching the transition states (TS) [10]. The Brillouin zone was sampled using a Gamma point grid due to the huge system of zeolite [11]. The (101) facet of MFI structure was used to simulate the PDH process without confinement microenvironment. The Gibbs free energy changes (ΔG) and kinetics barriers (G_a) of the elementaries are calculated by the followed equation:

$$\Delta G = [E_{FS} + (E_{ZPE} - T\times S)_{FS}] - [E_{IS} + (Z_{PE} - T\times S)_{IS}] \quad (9)$$

$$G_a = [E_{TS} + (E_{ZPE} - T\times S)_{TS}] - [E_{IS} + (Z_{PE} - T\times S)_{IS}] \quad (10)$$

Where the E_{IS}, E_{TS} and E_{FS} are the electronic energies of initial state, transition state and final state, respectively. The E_{ZPE}. T and S are zero-point energy, temperature and entropy.

1.4 Evaluation of catalytic performance

PDH tests were performed in a quartz micro-reactor (*i.d.* = 6 mm). Before the tests, 0.2 g catalyst (sieved to 40~60 mesh) was pretreated in H_2 flowing (20 mL min^{-1}) at 580 ℃ for 30 min. Then, the pretreated sample was purged by Ar (20 mL min^{-1}). About 30 min later, 5%C_3H_8/Ar mixture was fed upon the catalyst. The products were detected by an on-line gas chromatograph (Agilent GC 6890N) equipped with a flame ionization detector (FID, CP-PoraPlotQ-HT capillary column) and a thermal conductivity detector (TCD, TDX-01 packed column). The carbon balance of the Cr-MFI catalysts during PDH is higher than 95%. The conversion, selectivity, and yield of hydrocarbons were calculated as follows:

$$C_3H_8 \text{ conversion } (\%) = \frac{\sum n_i \times A_i - n_i \times A_{C_3H_8}}{\sum n_i \times A_i} \times 100 \quad (1)$$

$$\text{Selectivity } (\%) = \frac{n_i \times A_i}{\sum n_i \times A_i - n_i \times A_{C_3H_8}} \times 100 \quad (2)$$

$$\text{Yield } (\%) = \frac{n_{C_3H_6} \times A_{C_3H_6}}{\sum n_i \times A_i - n_i \times A_{C_3H_8}} \times 100 \quad (3)$$

Wherein *i*, n_i and A_i are the hydrocarbon products in the effluent gas stream, the number of carbon atoms of component *i* and the corrected mole concentration for compounds *i*.

The propylene formation rates (*r*) of the catalysts are calculated as follows:

$$r\ (\text{mmol}_{C_3H_6}\ g_{cat}^{-1}\ h^{-1}) = \frac{\text{moles of product } C_3H_6 \text{ formed per hour}}{\text{weight of catalyst}} \times 100 \quad (4)$$

The calculated apparent turn over frequencies (TOFs) of the supported Cr catalysts are based on all the Cr atoms.

$$\text{Calculated TOF } (h^{-1}) = \frac{\text{moles of product } C_3H_6 \text{ formed per hour}}{\text{moles of Cr atoms}} \times 100 \quad (5)$$

The cycle stability of Cr-MFI sample was conducted in the conditions: 0.2 g catalyst, atmosphere pressure, 580 ℃, 5%C_3H_8/Ar. Each cycle consists of a PDH step at 580 ℃ for 180 min, followed by a treatment in air at 580 ℃ for 10 min. Then, the sample was purged by Ar during these steps.

The deactivation rates of the PDH catalysts were calculated:

$$k_{\mathrm{d}} = \frac{\ln\frac{100-C_{\mathrm{e}}}{C_{\mathrm{e}}} - \ln\frac{100-C_{\mathrm{s}}}{C_{\mathrm{s}}}}{t} \tag{6}$$

where k_{d} (h^{-1}) is the deactivation rate, C_s and C_e are propane conversions at the start and end of the experiments respectively. t (h) is the reaction time. The sample with low k_{d} value possesses high catalytic stability.

2. Supplementary Figures

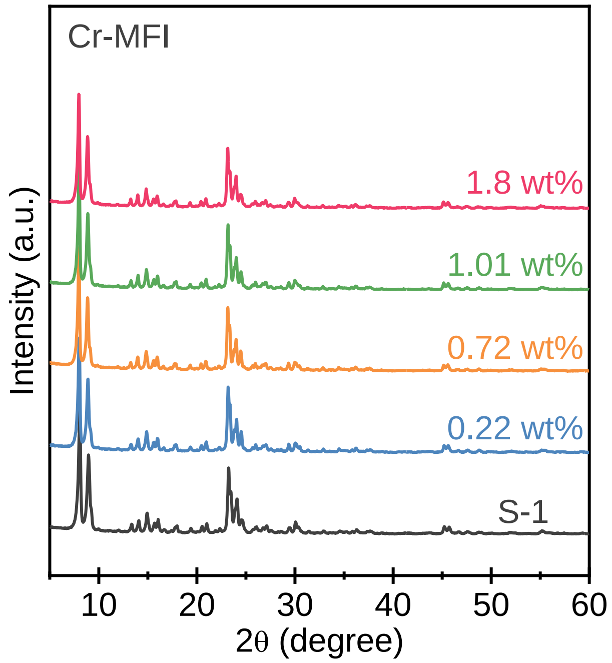

Figure S1. XRD patterns of S-1 and Cr-MFI with different Cr contents.

The XRD patterns of S-1 and Cr-MFI with different Cr contents show a series of diffraction peaks at 2θ = 6~10° and 12~32°, typical for the MFI zeolite framework. Note that no CrO_x peaks can be detected over the Cr-MFI zeolites, probably because the Cr species are highly dispersed onto MFI zeolite.

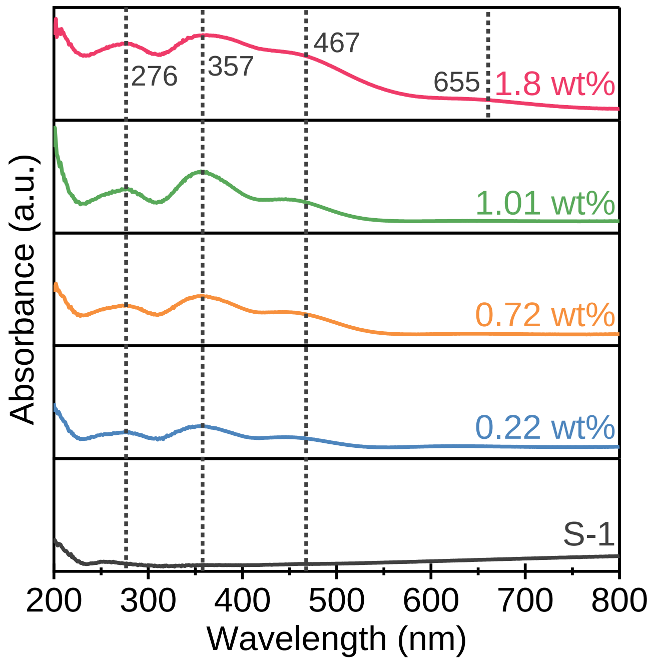

Figure S2. UV-vis spectra of S-1 and Cr-MFI with different Cr contents.

The UV-*vis* spectra of Cr-MFI catalysts possess three or four main peaks centered at the wavelengths of 276, 357, 467, and 655 nm, respectively. The bands at 276 and 357 nm correspond to the tetrahedral chromate transitions $^1T_2 \leftarrow {}^1A_1$ ($1t_1 \rightarrow 7t_2$ and $6t_2 \rightarrow 2e$) and $^1T_2 \leftarrow {}^1A_1$ ($1t_1 \rightarrow 2e$), respectively, and the bands at 467 and 655 nm are attributed to the Cr(VI) transition $^1T_1 \leftarrow {}^1A_1$ ($1t_1 \rightarrow 2e$) and octahedral symmetry $T_{2g} \leftarrow A_{2g}$ transition in α-Cr_2O_3 clusters, respectively [12−14]. At Cr content lower than 1.01 wt%, only three bands at 276, 357, and 467 nm are observed over the Cr-MFI, indicating the formation of only isolated Cr−O−Si species. With increasing Cr content to 1.8 wt%, CrO_x clusters or nanoparticles are formed upon the Cr-MFI.

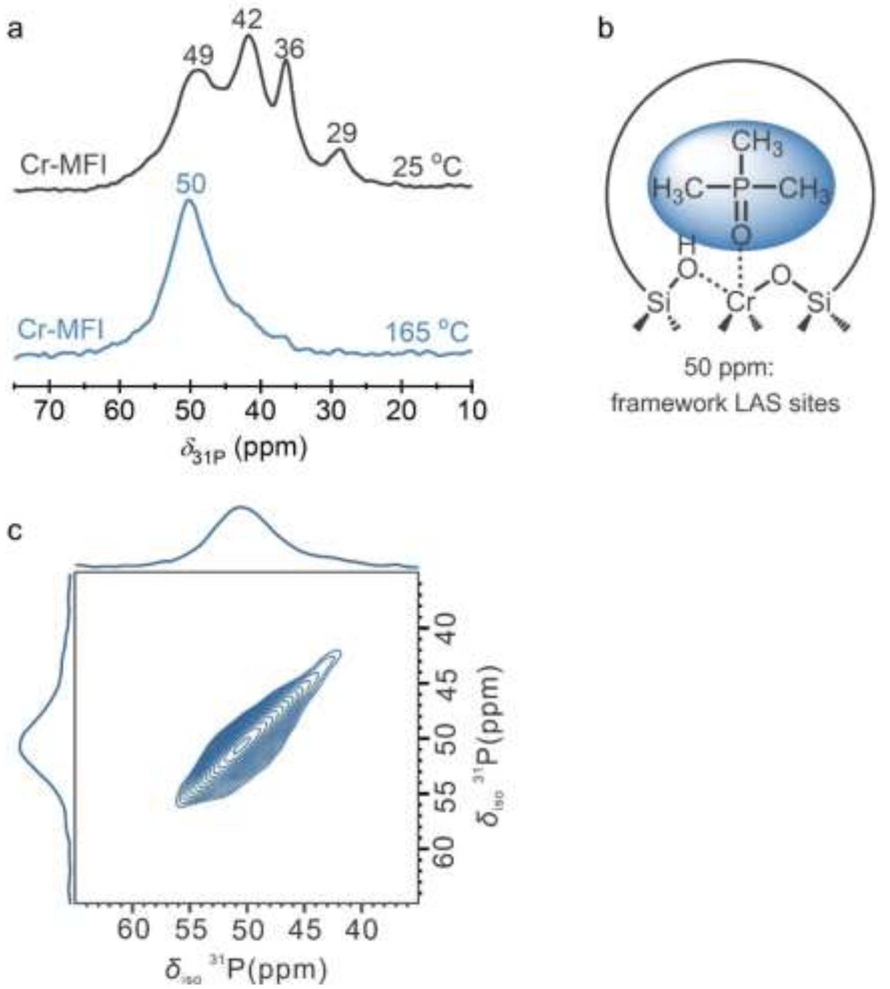

Figure S3. Solid-sate NMR spectra of Cr-MFI. (a) ^{31}P MAS NMR spectra of Cr-MFI after adsorption trimethylphosphine oxide (TMPO) and subsequently desorbed TMPO at 165 ℃. (b) Schematic illustration of TMPO adsorbed on the framework Cr sites of Cr-MFI. (c) ^{31}P-^{31}P MAS RFDR NMR spectra of Cr-MFI after adsorption TMPO and subsequently desorbed TMPO at 165 ℃ under ^{1}H spinal-64 decoupling.

The ^{31}P MAS NMR spectrum of Cr-MFI after saturation TMPO at room temperature has four prominent peaks at 29 ppm (physisorbed TMPO), 36 ppm (physisorbed TMPO in small pores), 42 ppm (crystalline TMPO), and 49 ppm (TMPO adsorbed on silanols and framework Cr sites), respectively (**Figure S3a**) [15]. After desorption at 165 ℃, only one peak at ~50 ppm is observed, corresponding to the TMPO interacting with framework Cr atoms (**Figure S3a**) [16,17]. The corresponding 2D spectra show no correlation (**Figure S3c**), confirming the homogeneous incorporation of Cr species into the MFI zeolite framework.

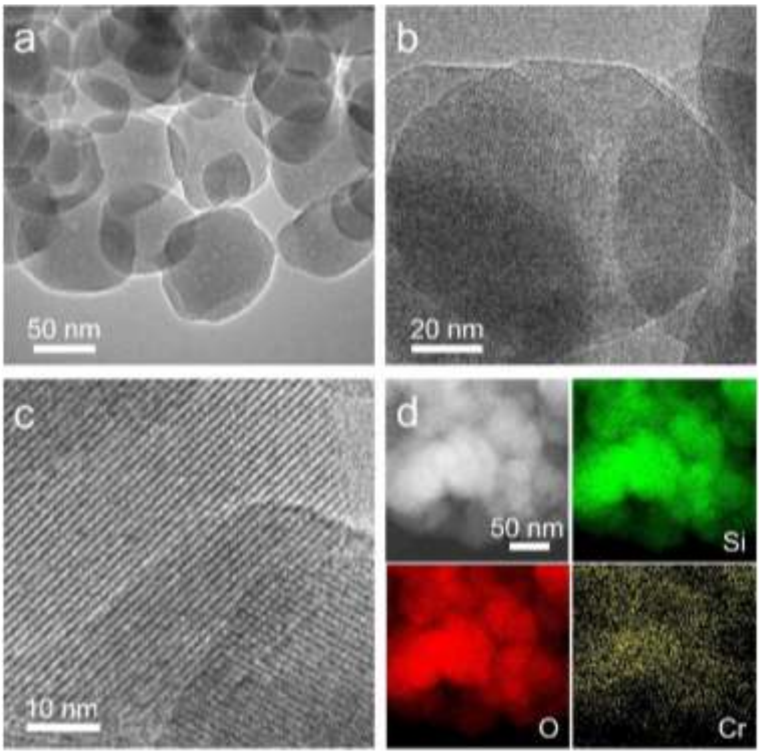

Figure S4. TEM images of Cr-MFI. (a-c)TEM. (d) STEM and EDS elemental mapping images of Cr-MFI.

The TEM images of Cr-MFI are composited of uniform cubes with the size of ~100 nm and possess very well-resolved lattice fringes. No CrO_x clusters and nanoparticles can be observed over the Cr-MFI. STEM-EDS mapping results reveal that the Cr species are homogeneously dispersed upon the MFI zeolite at the Cr loading of 1.01 wt%.

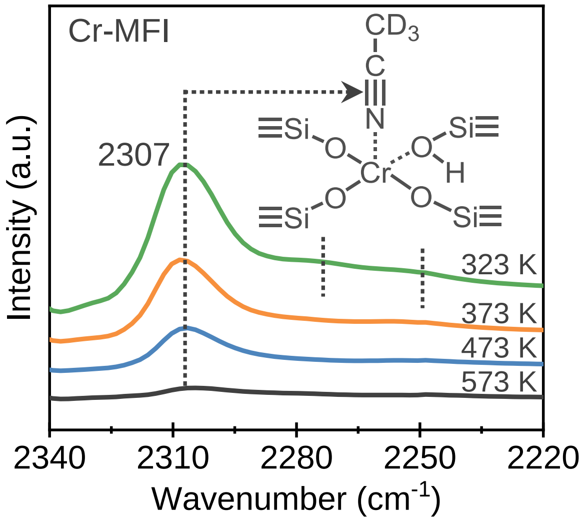

Figure S5. FTIR spectra of Cr-MFI after CD_3CN adsorption and desorption at different temperatures (323, 373, 473, and 573 K).

The FTIR spectra of Cr-MFI after CD_3CN adsorption and desorption show a strong peak at 2307 cm^{-1} and two very weak peaks at 2274 and 2253 cm^{-1}, which can be attributed to the v(C≡N) vision of CD_3CN adsorbed onto Cr Lewis acid site (2307 cm^{-1}) and silanol (2274 and 2253 cm^{-1}) [18,19]. Note that no other characteristic peaks about the open structures of Cr sites (*e.g.*, {$(\equiv SiO)_2CrOH(\equiv SiOH)$} and {$(\equiv SiO)_2Cr(OH)_2$}) can be observed, indicating the presence of a certain interactions between Cr and O atom in the Cr···OH−Si linkage.

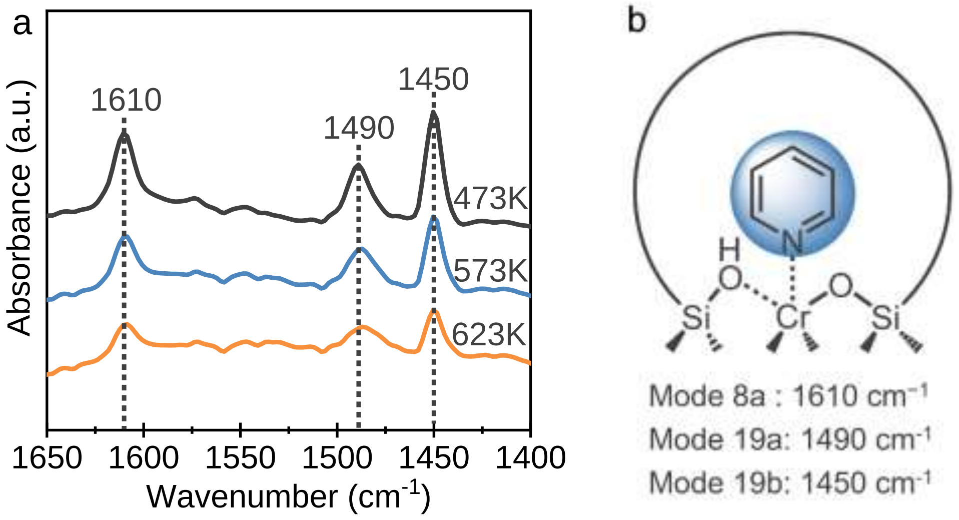

Figure S6. Pyridine-FTIR of Cr-MFI. a, FTIR spectra of Cr-MFI after pyridine adsorption and desorption at different temperatures (473, 573, and 623 K). b, Schematic illustration of the pyridine molecule adsorbed on Cr site of Cr-MFI.

It is known that the bands at 1,450, 1,490, and 1,610 cm^{-1} correspond to the Lewis acid sites (LAS), and the peaks at 1,490, 1,550, and 1,640 cm^{-1} are characteristic of Brønsted acid sites (BAS) [18,20]. As shown in Figure S6, all the spectra of Cr-MFI sample possess three bands at 1,450, 1,490, and 1,610 cm^{-1} without any signals at 1,550 and 1,640 cm^{-1}, suggesting the incorporation of Cr species into MFI zeolite framework only results in LAS.

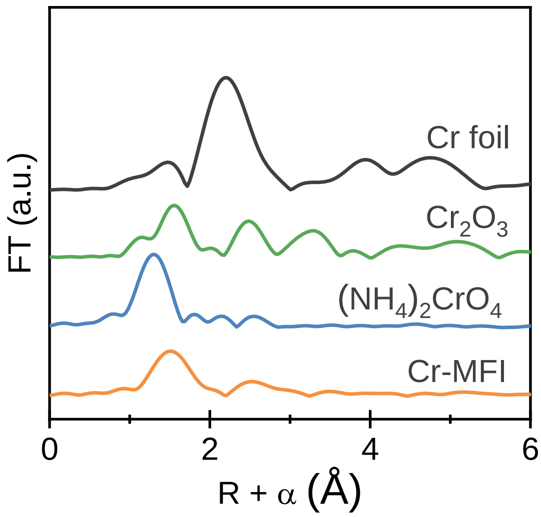

Figure S7. FT k^2-weighted EXAFS spectra of Cr foil, Cr_2O_3, $(NH_4)_2CrO_4$, and Cr-MFI.

The Fourier transform (FT) k^2-weighted extended X-ray absorption fine structure (EXAFS) spectrum of Cr-MFI exhibits two bands at 1.5 and 2.52 Å, which are different to Cr foil (2.24 Å, metallic Cr−Cr) and Cr_2O_3 (1.54, Cr−O; 2.55 and 3.3 Å, Cr−O−Cr), and can be attributed to the Cr−O (1.5 Å) and Cr−O−Si (2.52 Å) species, respectively.

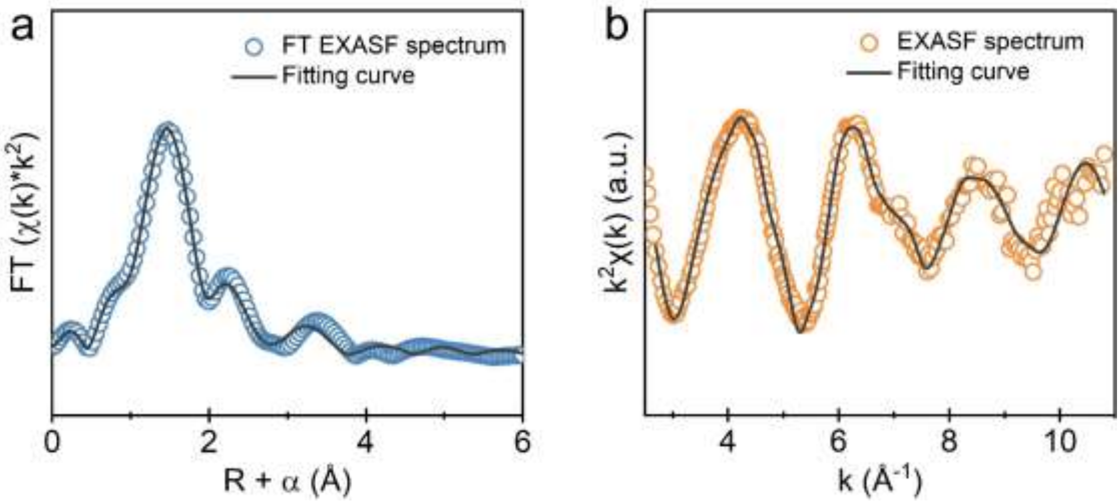

Figure S8. Cr K-edge EXAFS (circle) and fitting curve (line) for Cr-MFI. R-space (a) and k-space (b).

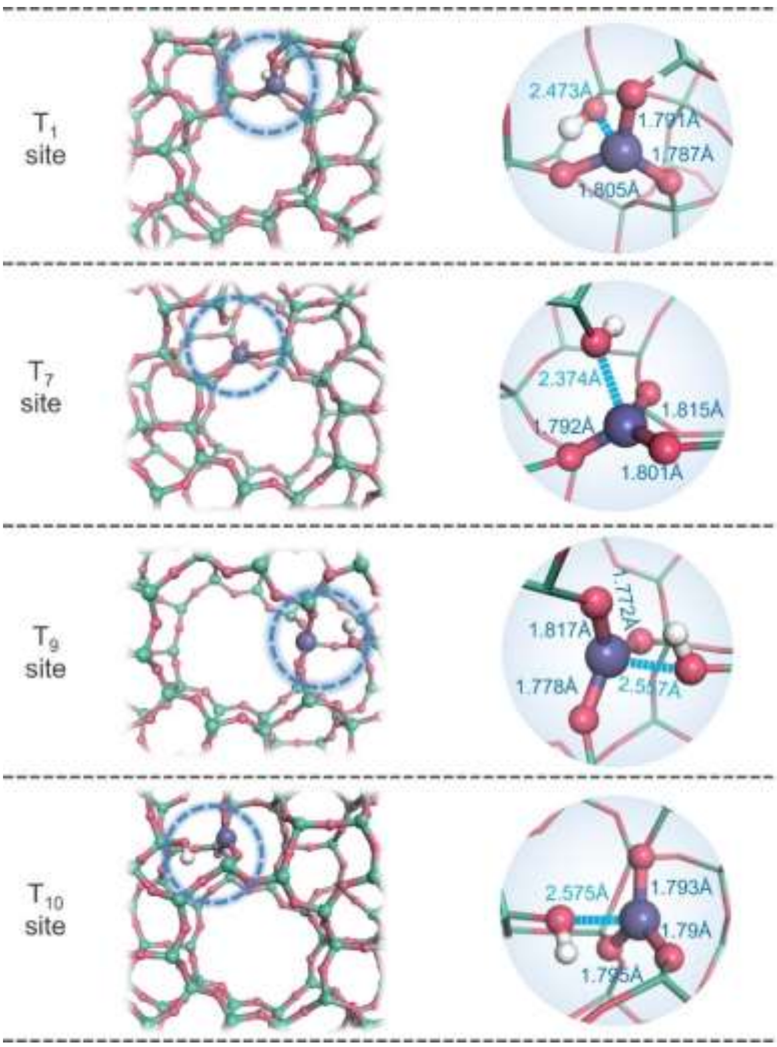

Figure S9. Local structure of Cr-MFI with Cr at T_1, T_7, T_9, and T_{10} sites by DFT simulation.

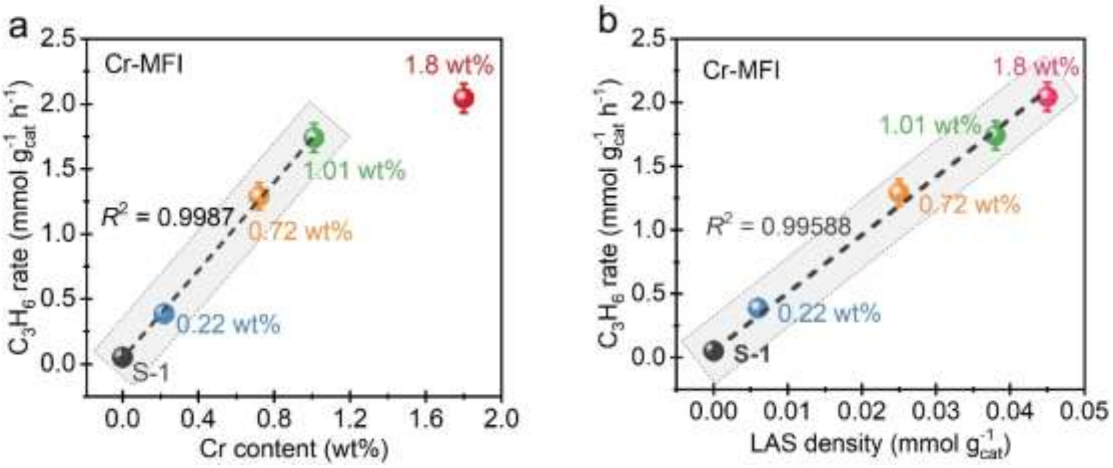

Figure S10. (a) Initial propylene formation rates of Cr-MFI catalysts with different Cr contents. (b) Propylene formation rate plotted as the density of Lewis acid sites (LAS) of Cr-MFI samples.

The initial catalytic activities of Cr-MFI catalysts show a strong dependence on Cr content (**Figure S10a**). At low Cr loading (0~1.01 wt%), the propylene formation rates of Cr-MFI catalysts exhibit a linear relationship with Cr content. However, this correlation disappears in Cr-MFI with 1.8 wt% Cr loading. Notably, the propylene formation rates demonstrate an excellent linear relationship with LAS density (**Figure S10b**), indicating that LAS derived from Cr species are responsible for propylene formation during the PDH reaction.

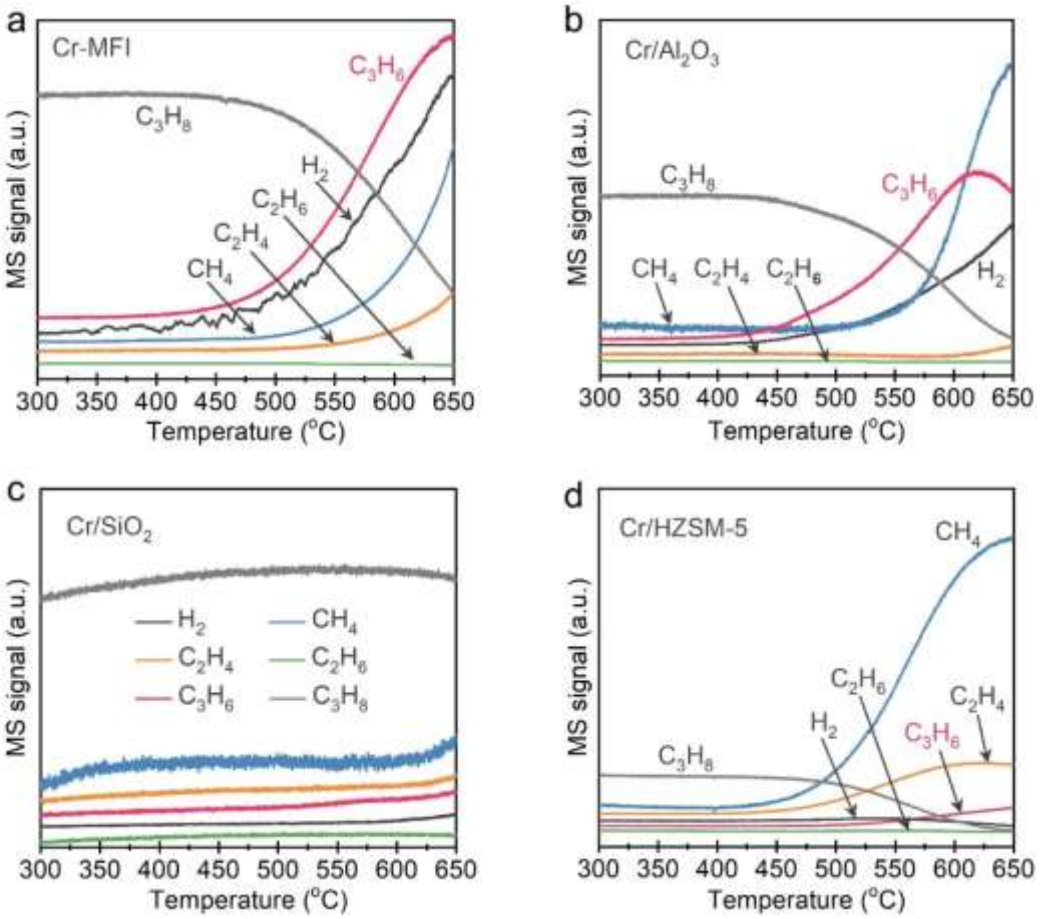

Figure S11. TPSR results of different Cr-based catalysts. (a) Cr-MFI, (b) Cr/Al_2O_3, (c) Cr/SiO_2, (d) Cr/HZSM-5.

The PDH properties of Cr-MFI and three other contrast catalysts (Cr/SiO_2, Cr/Al_2O_3, and Cr/HZSM-5) are examined by C_3H_8-TPSR experiments. As to Cr-MFI, the C_3H_8 signal starts to decrease at 450 □ with the C_3H_6 and H_2 signals increasing. Particularly, the C_3H_6 and H_2 signals increase obviously with only a slight increase of CH_4, C_2H_4, and C_2H_6 signals in the temperature range of 450~600 □. Further increasing the temperature to 650 □ would result in a great increase of the CH_4 and C_2H_4 signals, suggesting the high temperature leads to cracking reaction ($C_3H_8 \rightarrow CH_4 + C_2H_4$). Differently, the Cr/$SiO_2$ sample shows very low H_2, CH_4, C_2H_4, C_2H_6, and C_3H_6 signals and a stable C_3H_8 signal in the temperature range of 300~600 □, indicating the very low PDH performance. For Cr/Al_2O_3, it exhibits a higher CH_4 signal than that of Cr-MFI, in particular at high reaction temperature, suggesting the presence of serious cracking reaction on Cr/Al_2O_3 catalyst. The TPSR results demonstrate the excellent PDH performance of the Cr-MFI catalyst.

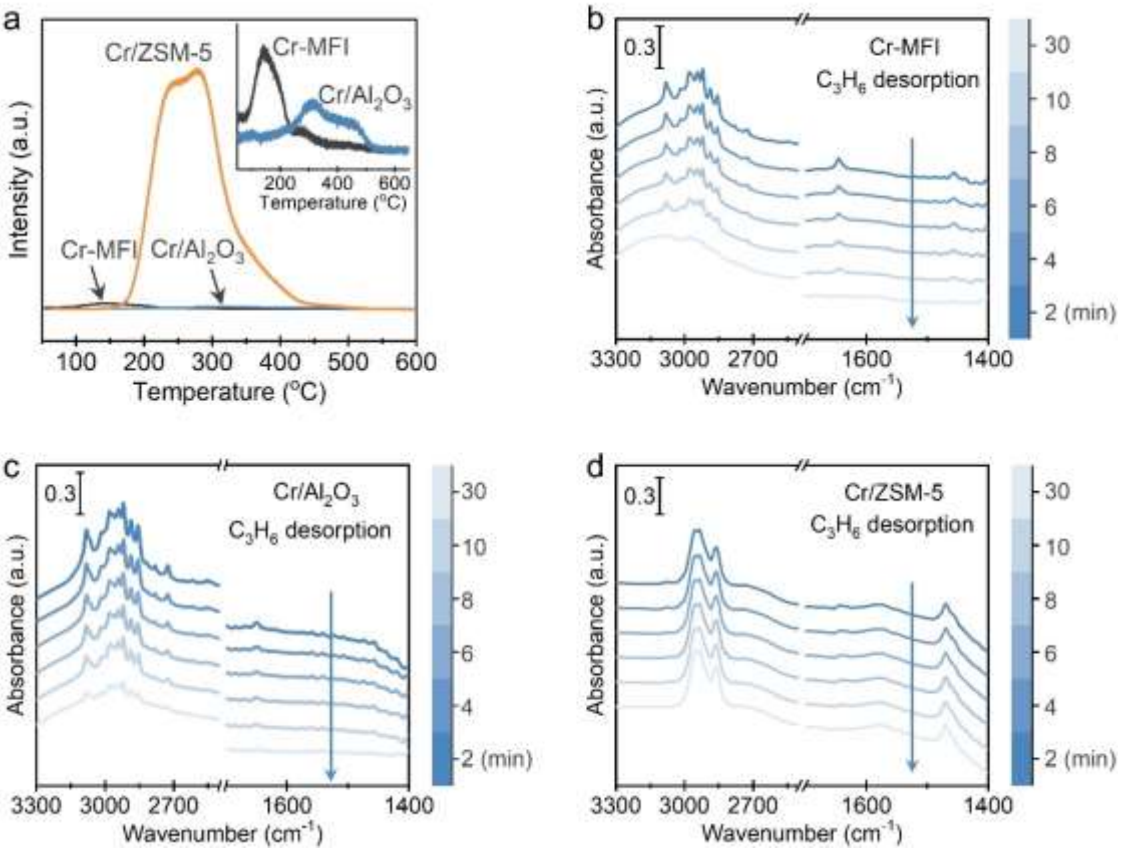

Figure S12. C_3H_6-TPD analysis of Cr-based catalysts. (a) C_3H_6-TPD results of Cr-MFI, Cr/Al_2O_3, and Cr/HZSM-5. (b) C_3H_6-TPD-FTIR results of Cr-MFI. (c) C_3H_6-TPD-FTIR results of Cr/Al_2O_3. (d) C_3H_6-TPD-FTIR results of Cr/HZSM-5.

The C_3H_6-TPD curve Cr/HZSM-5 exhibits a much stronger propylene desorbed signal in the temperature range of 200 ~ 500 □ than that of the Cr-MFI and Cr/Al_2O_3. The enlarged curves show that the propylene desorption temperature of Cr-MFI (centered at ~170 □) is much lower than that of Cr/Al_2O_3 (200~500 □), indicating the presence of weak interactions between propylene and Cr-MFI catalysts.

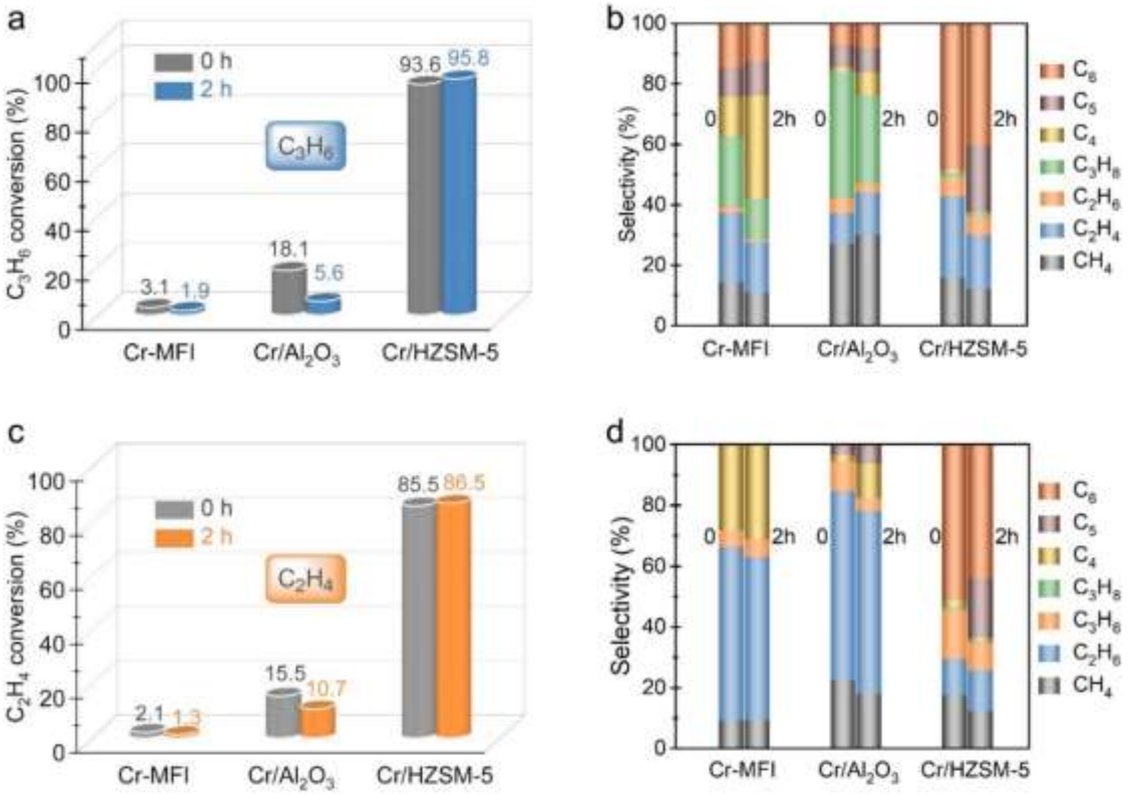

Figure S13. Catalytic results of Cr-MFI, Cr/Al$_2$O$_3$, and Cr/HZSM-5. Catalytic results of Cr-MFI, Cr/Al$_2$O$_3$, and Cr/HZSM-5 at the reaction conditions of (a, b) 5%C_3H_6/N_2, 580 □, 0.2 g catalyst, and WHSV = 0.6 h^{-1} and (c, d) 5%C_2H_4/N_2, 600 □, 0.2 g catalyst, and WHSV = 0.4 h^{-1}. (a) C_3H_6 conversion. (b) CH_4, C_2H_4, C_2H_6, C_3H_8, C_4, C_5, and C_6 selectivity at 0 and 2 h, respectively. (c) C_2H_4 conversion. (d) CH_4, C_2H_6, C_3H_6, C_3H_8, C_4, C_5, and C_6 selectivity at 0 and 2 h, respectively.

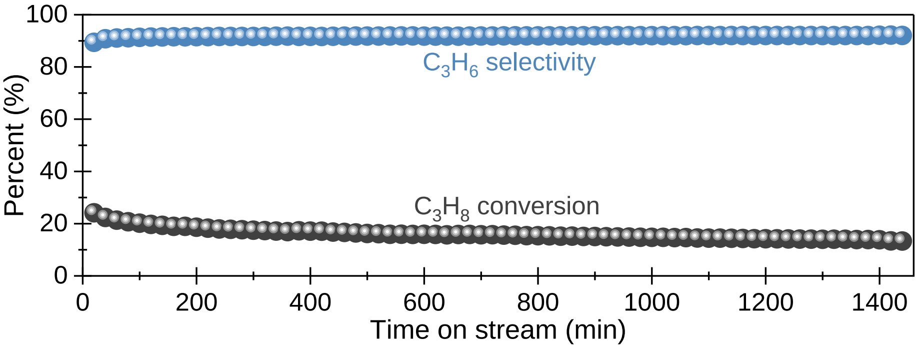

Figure S14. Propane conversion and propylene selectivity of Cr-MFI. Reaction conditions: 0.2 g catalyst, 580 □, WHSV = 2.4 h^{-1}.

The Cr-MFI catalyst was tested at two different weight hourly space velocity (WHSV) conditions. At the WHSV of 0.6 h^{-1}, Cr-MFI catalyst exhibits a very high PDH performance, *i.e.*, the initial propylene yield and selectivity of Cr-MFI are ~40.6% and ~93.0%, respectively. After 24 h time on stream, ~28.3% propane conversion and ~94.0% propylene selectivity can be obtained. With increasing the WHSV to 2.4 h^{-1}, the propane conversion and propylene decrease to 24.2% and 89.4%, respectively. After 24 h time on stream, ~13.3% propane conversion and ~92.1% propylene selectivity can be still maintained. Noticeably, the propylene formation rate of Cr-MFI increases accompanied by a drop in conversion and selectivity with increasing the WHSV, indicating more severe cracking and deep dehydrogenation reactions.

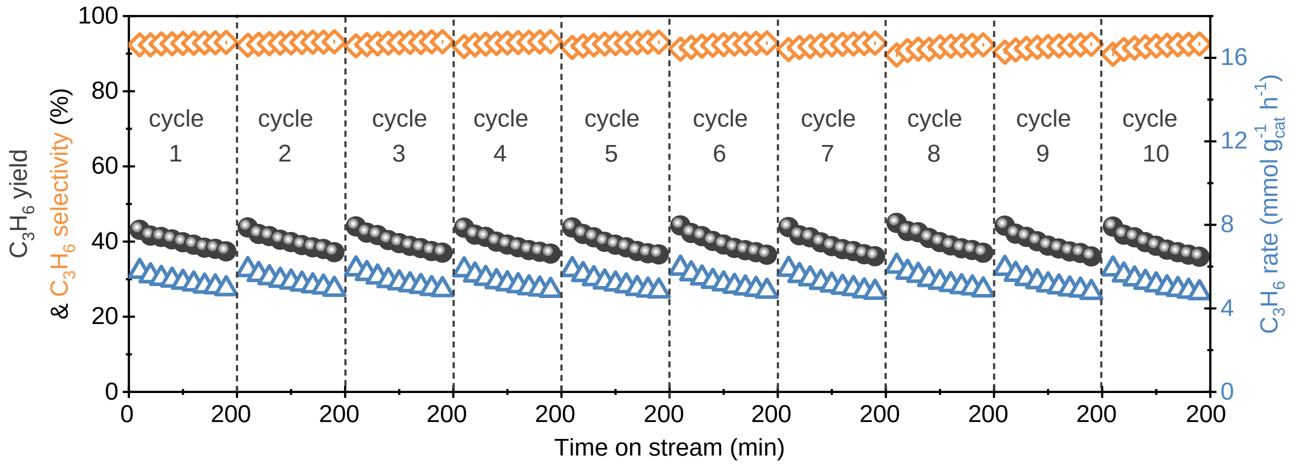

Figure S15. Cycle stability of the Cr-MFI. Regeneration conditions: 580 □, air (20 mL min^{-1}), 10 min, WHSV = 0.6 h^{-1}.

The cycle testing results of Cr-MFI show an initial propylene formation rate of 5.78 mmol g_{cat}^{-1} h^{-1} in the first cycle and 5.89 mmol g_{cat}^{-1} h^{-1} after 10th regeneration. These results suggest that the original activity of Cr-MFI can be fully recovered, demonstrating the excellent catalytic stability of Cr-MFI catalysts.

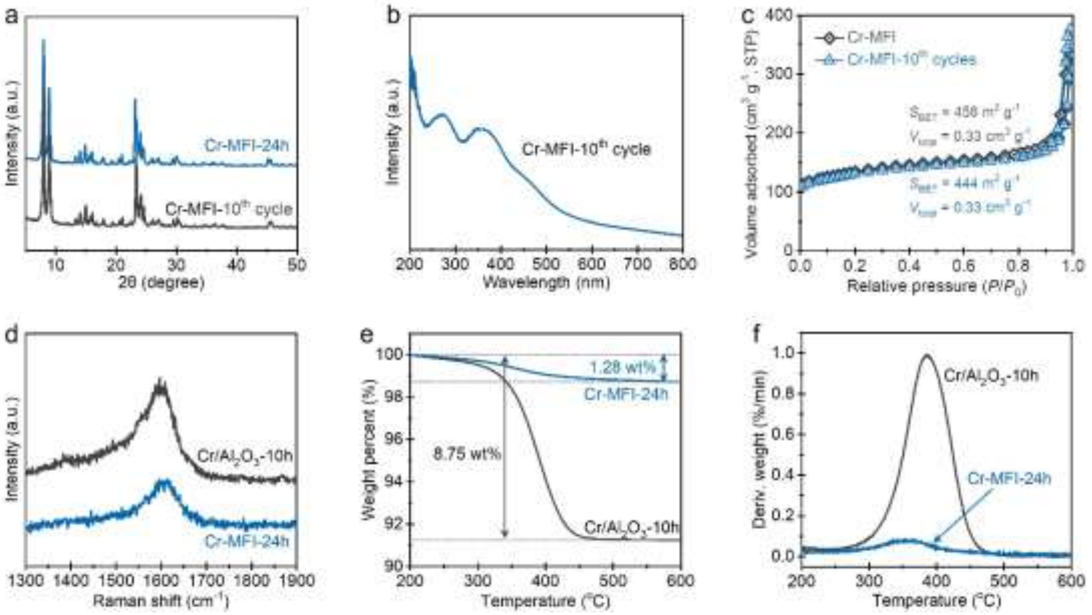

Figure S16. Characterizations of the spent Cr-MFI catalysts: (a) XRD patterns of Cr-MFI after 24 h time on stream and 10th cycle. (b) UV-vis spectrum of Cr-MFI after 10th cycle. (c) N_2 adsorption-desorption isotherms of Cr-MFI before and after 10th cycle. (d) Raman spectra of Cr-MFI after 24 h time on stream and Cr/Al_2O_3 after 10 h time on stream. (e) TG curves and (f) the corresponding derive weight curve of Cr-MFI after 24 h time on stream and Cr/Al_2O_3 after 10 h time on stream.

Characterization of spent Cr-MFI catalysts was performed using XRD, UV-vis, N_2 sorption, Raman spectroscopy, and TG analysis. The XRD patterns of the spent Cr-MFI catalyst after 24 h time-on-stream (TOS) and 10 reaction cycles maintain well-defined MFI structural features (**Figure S16a**). UV-vis spectra (**Figure S16b**) and N_2 sorption isotherms (**Figure S16c**) of the cycled Cr-MFI closely resemble those of the fresh catalyst, confirming its exceptional thermal stability. Raman analysis reveals significantly higher coke deposition on spent 1%Cr/Al_2O_3 compared to Cr-MFI (**Figure S16d**), further supported by TG results: 1%Cr/Al_2O_3 exhibits an 8.75% weight loss after 10 h TOS, whereas Cr-MFI shows only a minimal 1.28% loss even after 24 h TOS (**Figure S16e** and **S16f**). These results collectively demonstrate Cr-MFI's superior anti-coking capability in PDH reactions.

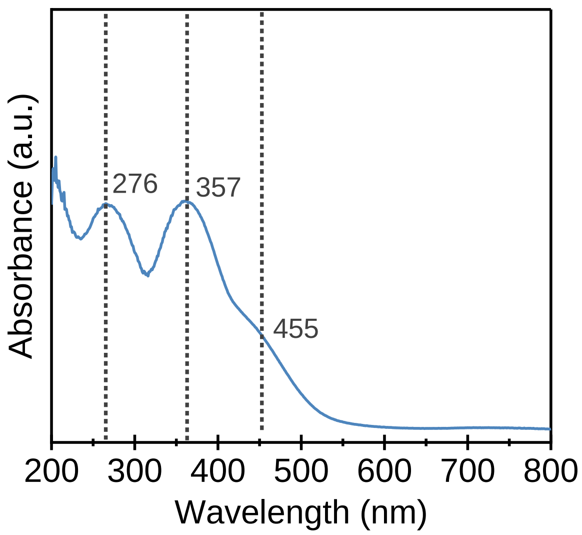

Figure S17. UV-vis spectra of Cr/SiO_2 with Cr content of 1 wt%.

The UV-*vis* spectrum of Cr/SiO_2 catalyst possesses three main peaks centered at the wavelength of 276, 357, and 455 nm, respectively, which are similar to the those of Cr-MFI. The absence of the band at 655 nm indicates the formation of homogeneous isolated Cr site attached onto the SiO_2 support.

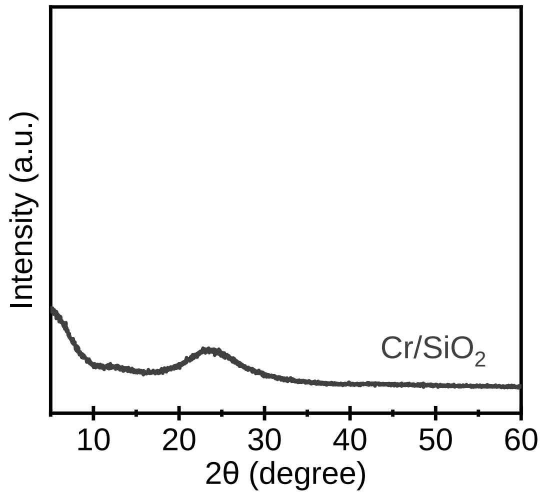

Figure S18. XRD patterns of Cr/SiO_2.

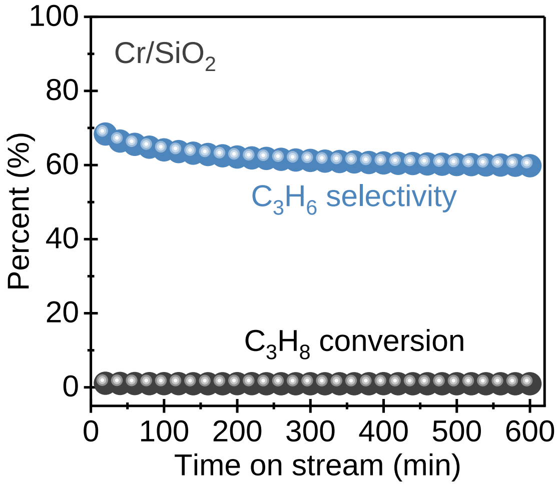

Figure S19. Propane conversion and propylene selectivity of Cr/SiO_2. Reaction conditions: 0.2 g catalyst, WHSV = 0.6 h^{-1}, 580 □.

The PDH result of Cr/SiO_2 exhibits very low PDH performance with an initial propane conversion of 1.1% and a propylene selectivity of 68.4%. This suggests the type of supports plays an important role in determining the PDH performance of Cr-based catalysts.

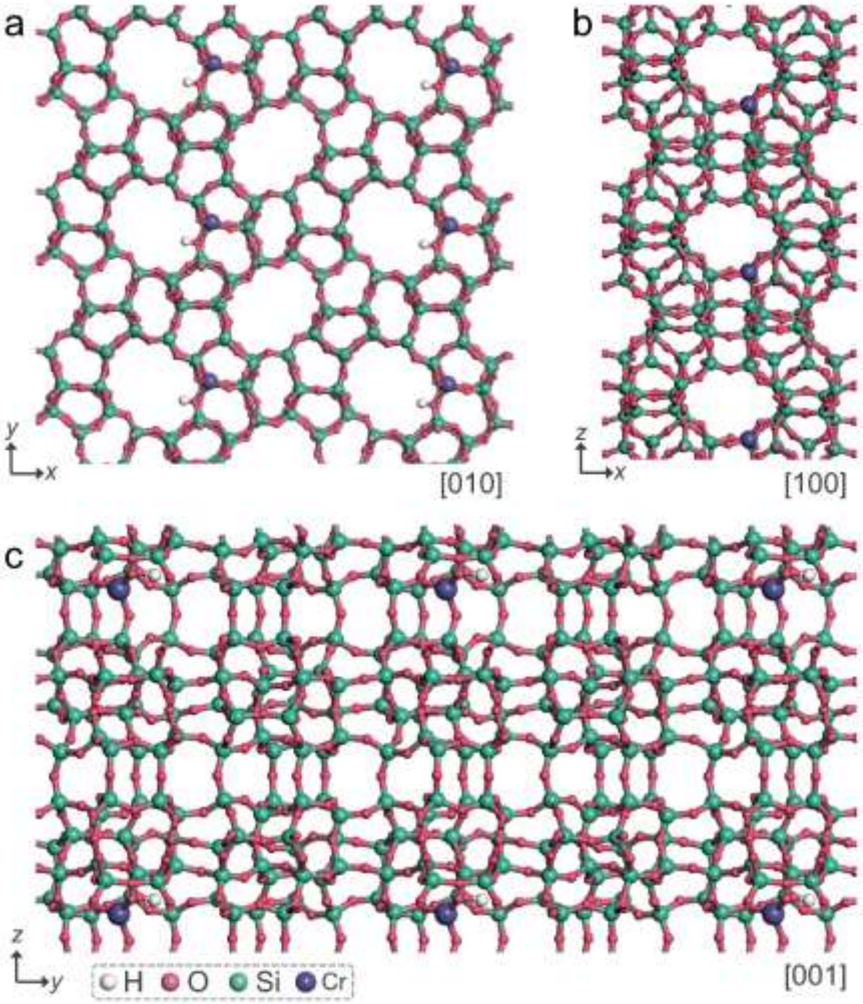

Figure S20. Local structure of Cr-MFI with Cr at T_3 site with complete pore environment by DFT simulation.

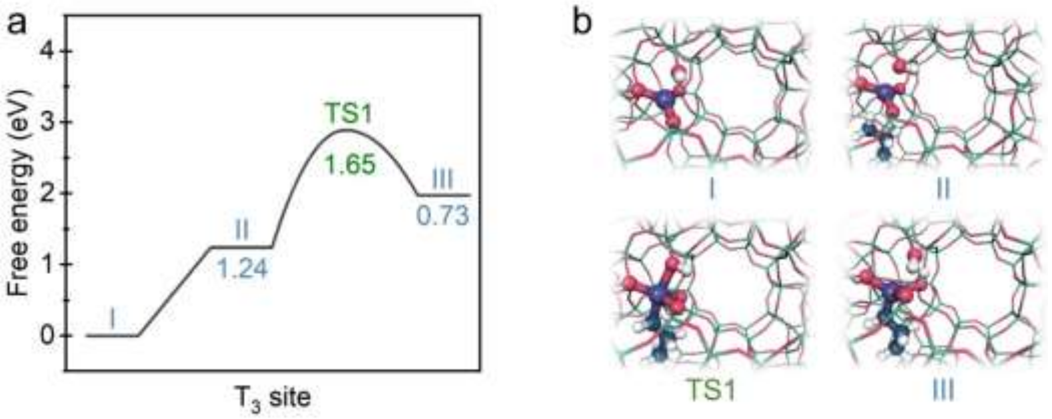

c

	d(Cr-C) Å	d(Cr···O) Å	d(Cr-O) Å	d(Cr-H) Å
I	–	2.404	1.8, 1.806, 1.809	–
II	3.44	2.398	1.8, 1.803, 1.808	–
TS1	2.223	2.274	1.839, 1.871, 2.04	–
III	2.022	2.261, 2.939	1.852, 1.867	–

Figure S21. DFT calculations of the first C−H bond dissociation of C_3H_8 on Cr-MFI with Cr atom locating at the T_3 site with complete microenvironment. (a) free energy. (b) optimized structures. (c) DFT predicted the Cr−C, Cr−O, and Cr···O.

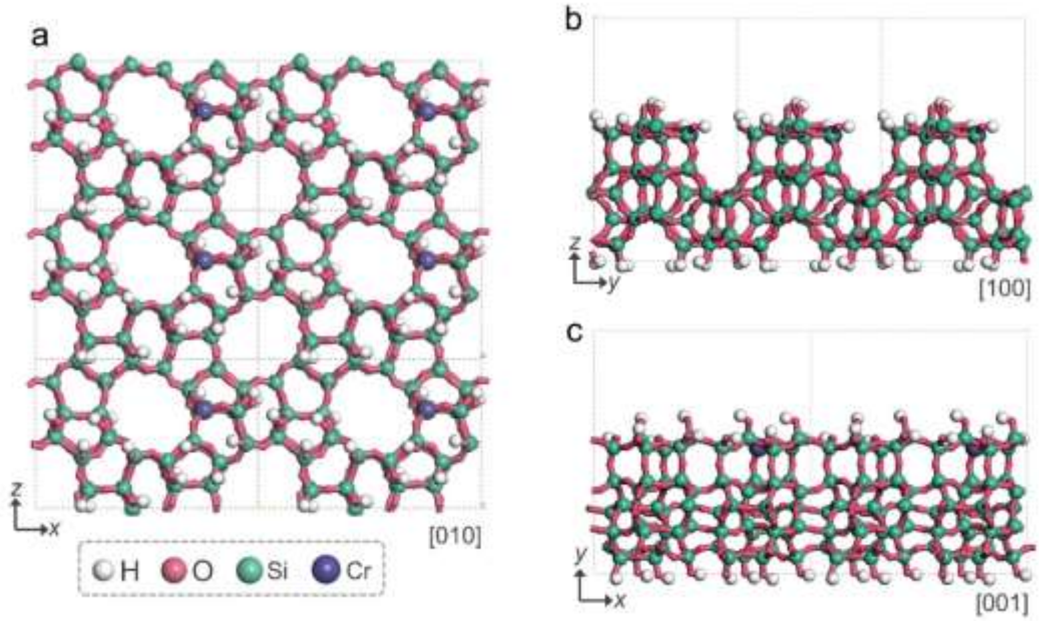

Figure S22. Local structure of Cr-MFI with Cr at T_3 site without microenvironment by DFT simulation.

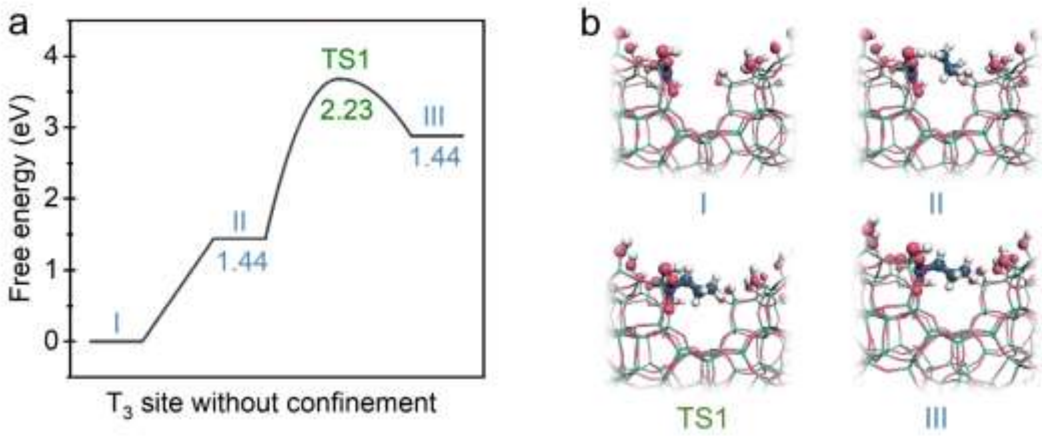

c

	d(Cr-C) Å	d(Cr···O) Å	d(Cr-O) Å	d(Cr-H) Å
I	–	–	1.799, 1.801, 1.814	–
II	3.489	–	1.8, 1.801, 1.814	–
TS1	2.183	–	1.841, 1.858, 2.0	–
III	2.020	2.555	1.824, 1.847	–

Figure S23. DFT calculations of the first C−H bond dissociation of C_3H_8 on Cr-MFI with Cr atom locating at the T_3 site without complete microenvironment. (a) free energy. (b) optimized structures. (c) DFT predicted the Cr−C, Cr−O, and Cr···O.

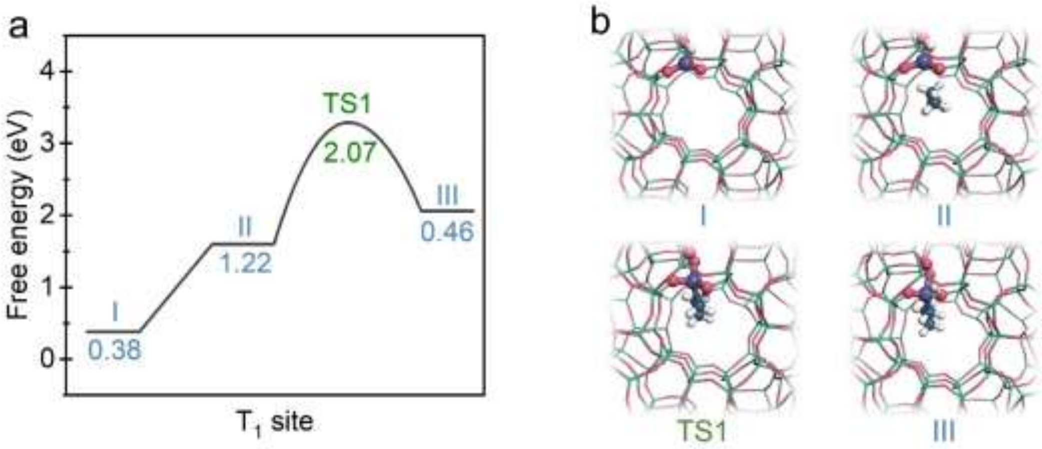

c

	d(Cr-C) Å	d(Cr···O) Å	d(Cr-O) Å	d(Cr-H) Å
I	–	2.473	1.787, 1.791, 1.805	–
II	3.834	2.524	1.783, 1.785, 1.805	–
TS1	2.184	3.151	1.833, 1.868, 1.946	–
III	2.018	2.347, 3.382	1.825, 1.862	–

Figure S24. DFT calculations of the first C−H bond dissociation of C_3H_8 on Cr-MFI with Cr atom locating at the T_1 site with complete microenvironment. (a) free energy. (b) optimized structures. (c) DFT predicted the Cr−C, Cr−O, and Cr···O.

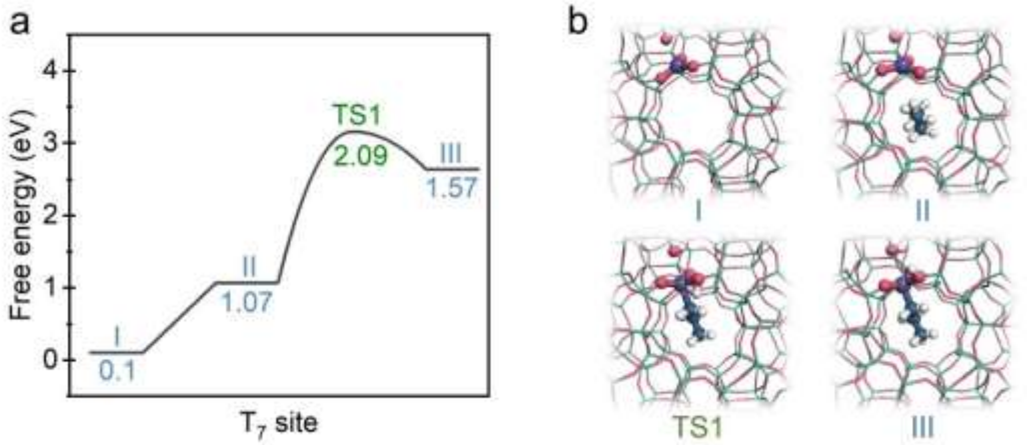

c

	d(Cr-C) Å	d(Cr···O) Å	d(Cr-O) Å	d(Cr-H) Å
I	–	2.374	1.792, 1.801, 1.815	–
II	4.273	2.314	1.802, 1.805, 1.809	–
TS1	2.288	2.709	1.853, 1.913, 1.960	–
III	2.066	2.041, 2.859	1.910, 1.943	–

Figure S25. DFT calculations of the first C−H bond dissociation of C_3H_8 on Cr-MFI with Cr atom locating at the T_7 site with complete microenvironment. (a) free energy. (b) optimized structures. (c) DFT predicted the Cr−C, Cr−O, and Cr···O.

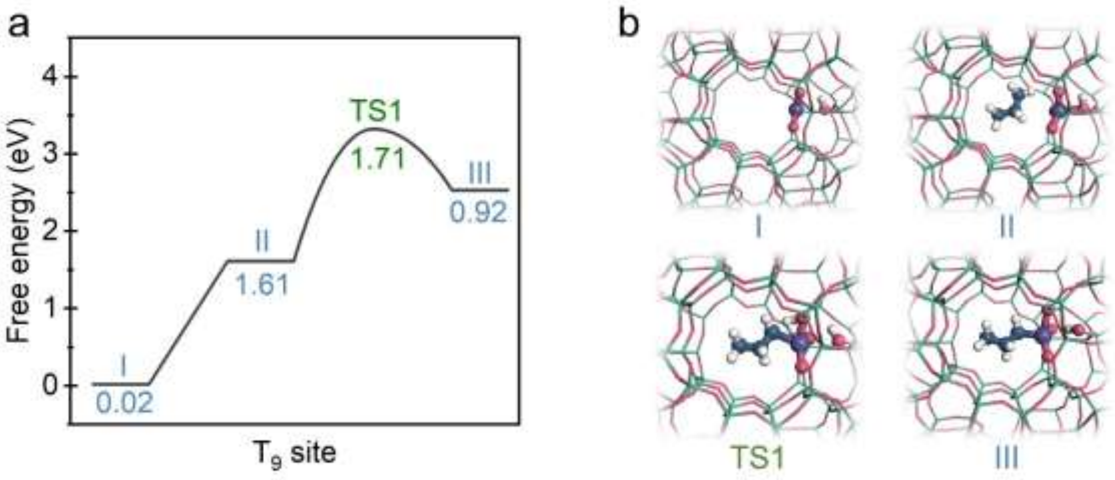

c

	d(Cr-C) Å	*d*(Cr···O) Å	*d*(Cr-O) Å	*d*(Cr-H) Å
I	–	2.557	1.772, 1.778, 1.817	–
II	3.850	2.064	1.789, 1.793, 1.849	–
TS1	2.194	2.783	1.818, 1.854, 1.990	–
III	2.016	2.164, 2.850	1.816, 1.899	–

Figure S26. DFT calculations of the first C−H bond dissociation of C_3H_8 on Cr-MFI with Cr atom locating at the T_9 site with complete microenvironment. (a) free energy. (b) optimized structures. (c) DFT predicted the Cr−C, Cr−O, and Cr···O.

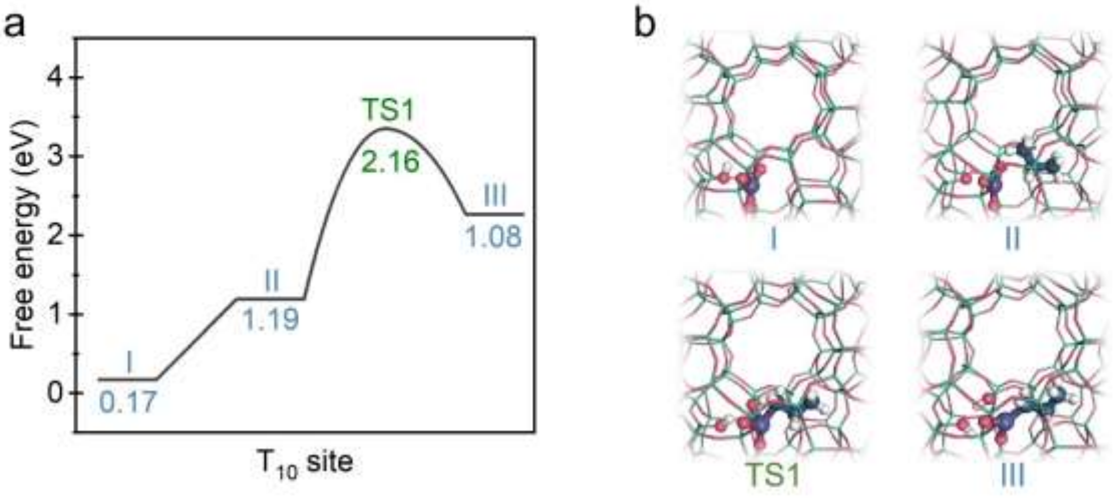

c

	d(Cr-C) Å	d(Cr···O) Å	d(Cr-O) Å	d(Cr-H) Å
I	–	2.575	1.790, 1.793, 1.795	–
II	4.103	2.630	1.790, 1.790, 1.795	–
TS1	2.186	3.402	1.841, 1.862, 1.980	–
III	2.027	2.465, 3.504	1.827, 1.869	–

Figure S27. DFT calculations of the first C−H bond dissociation of C_3H_8 on Cr-MFI with Cr atom locating at the T_{10} site with complete microenvironment. (a) free energy. (b) optimized structures. (c) DFT predicted the Cr−C, Cr−O, and Cr···O.

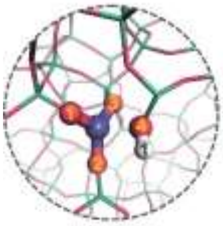

I	charge		charge
O_1	-1.31	H	+0.68
O_2	-1.32	Cr	+1.59
O_3	-1.30		
O_4	-1.45		

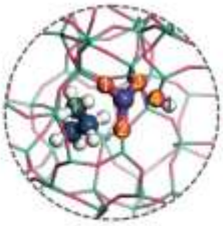

II	charge		charge
O_1	-1.31	C	+0.01
O_2	-1.32	H	+0.78
O_3	-1.32	Cr	+1.59
O_4	-1.55		

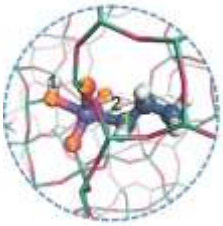

TS1	charge		charge
O_1	-1.36	C	-0.40
O_2	-1.35	H_1	+0.73
O_3	-1.33	H_2	+0.45
O_4	-1.49	Cr	+1.54

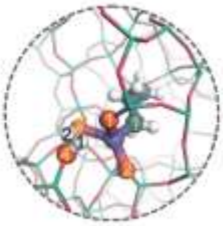

III	charge		charge
O_1	-1.37	C	-0.37
O_2	-1.37	H_1	+0.72
O_3	-1.47	H_2	+0.70
O_4	-1.52	Cr	+1.46

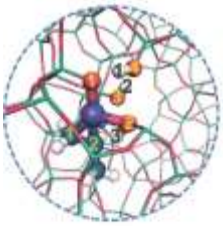

TS2	charge		charge
O_1	-1.43	C_2	-0.34
O_2	-1.37	H_1	+0.72
O_3	-1.46	H_2	+0.67
O_4	-1.51	H_3	+0.48
C_1	-0.29	Cr	+1.29

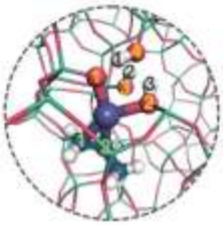

IV	charge		charge
O_1	-1.47	C_2	-0.24
O_2	-1.54	H_1	+0.68
O_3	-1.49	H_2	+0.69
O_4	-1.49	H_3	+0.80
C_1	-0.28	Cr	+0.99

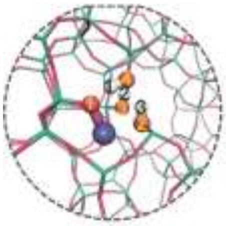

V	charge		charge
O_1	-1.46	H_1	+0.69
O_2	-1.45	H_2	+0.69
O_3	-1.49	H_3	+0.66
O_4	-1.51	Cr	+0.83

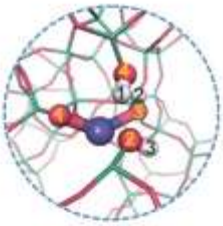

TS3	charge		charge
O_1	-1.48	H_1	+0.66
O_2	-1.45	H_2	+0.67
O_3	-1.47	H_3	+0.66
O_4	-1.47	Cr	+0.80

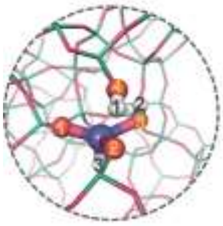

VI	charge		charge
O_1	-1.36	H_1	+0.67
O_2	-1.36	H_2	+0.72
O_3	-1.46	H_3	-0.31
O_4	-1.47	Cr	+1.46

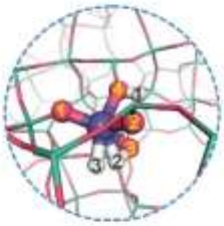

TS4	charge		charge
O_1	-1.37	H_1	+0.81
O_2	-1.33	H_2	+0.41
O_3	-1.33	H_3	-0.30
O_4	-1.55	Cr	+1.54

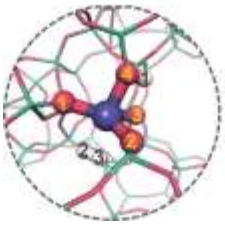

VII	charge		charge
O_1	-1.36	H_1	+0.70
O_2	-1.33	Cr	+1.64
O_3	-1.38		
O_4	-1.60		

Figure S28. The charges of H, C, O, and Cr atom at T_3 site of Cr-MFI during PDH reaction by DFT calculations.

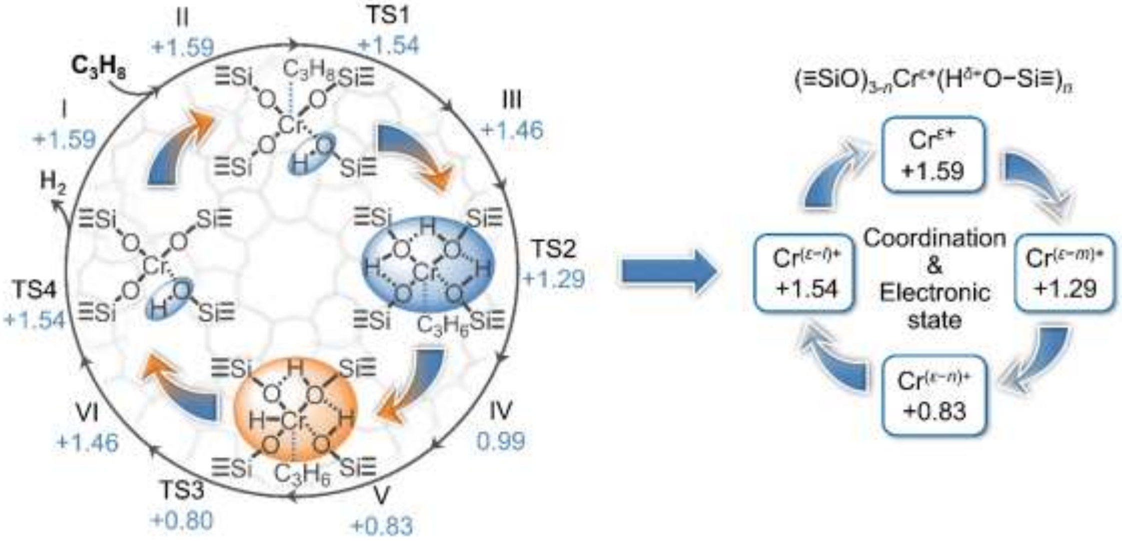

Figure S29. Dynamic evolution of the Cr electronic states during PDH reaction by DFT calculations.

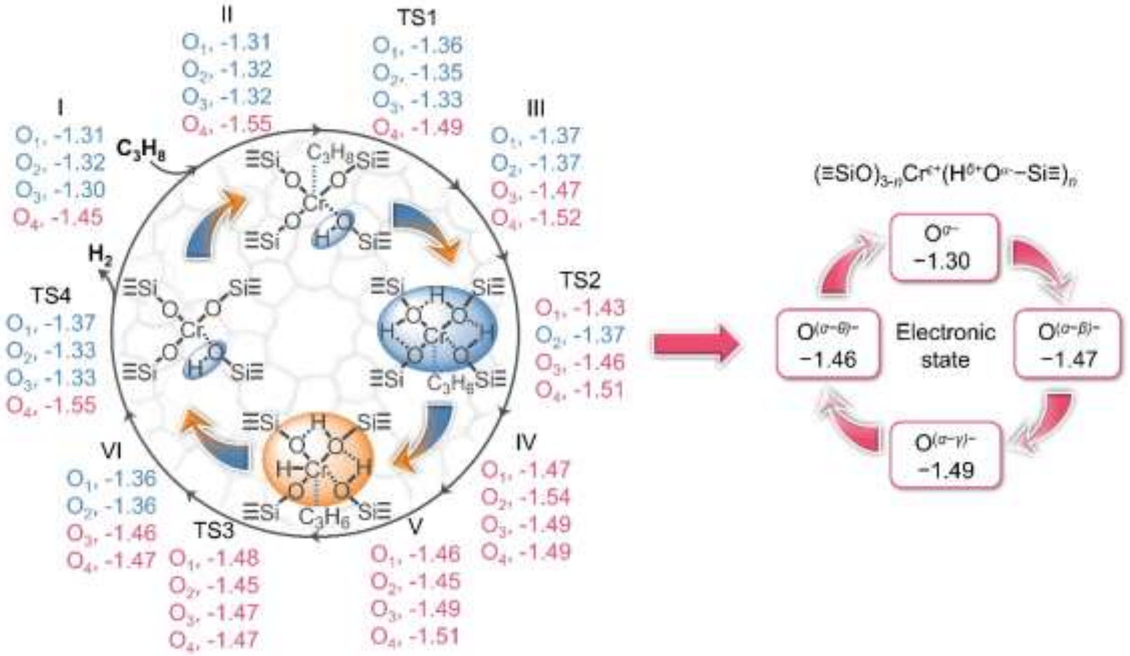

Figure S30. Dynamic evolution of the O electronic states during PDH reaction by DFT calculations.

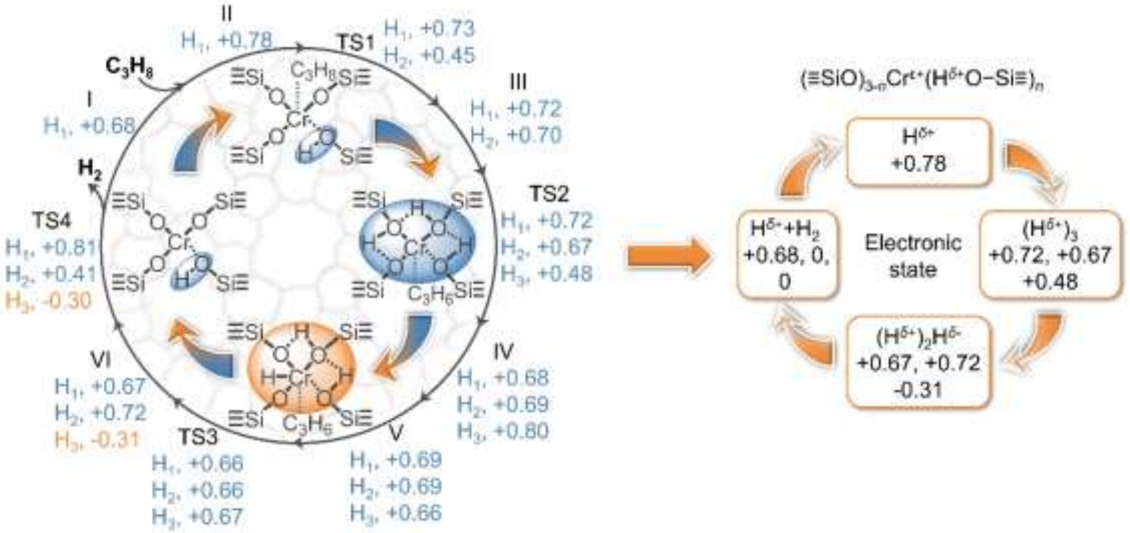

Figure S31. Dynamic evolution of the H electronic states during PDH reaction by DFT calculations.

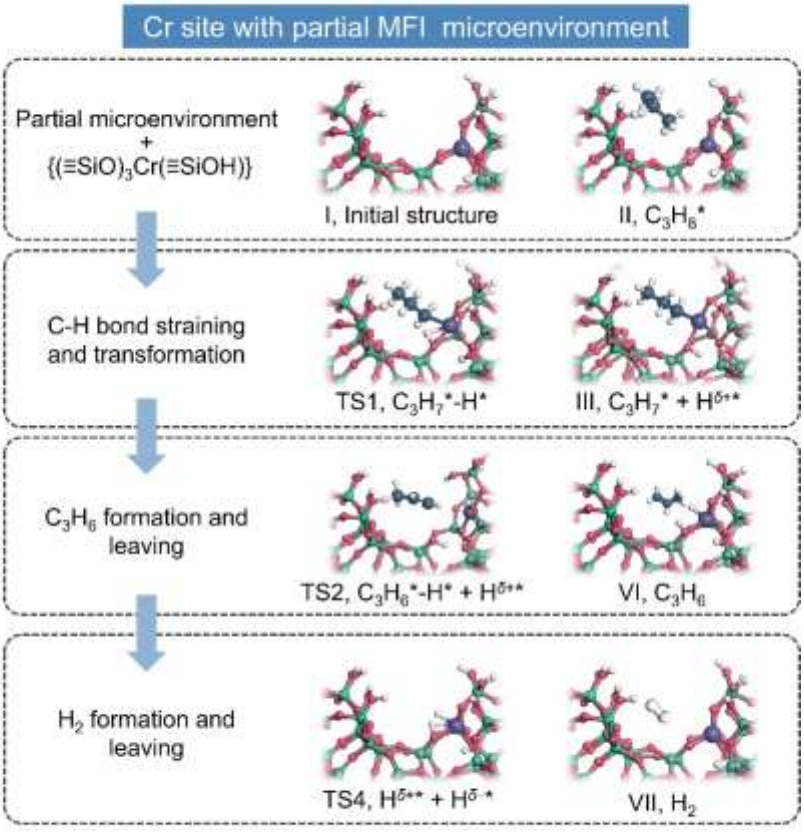

Figure S32. The corresponding structures at different steps over the Cr-MFI zeolite with partial microenvironment.

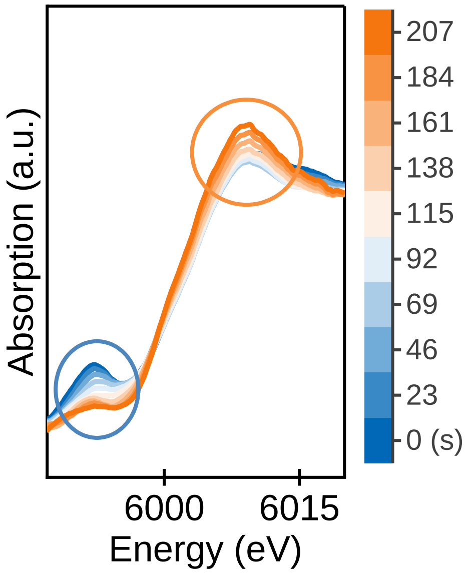

Figure S33. In situ XAS spectra of Cr-MFI pretreated in 10%H_2/He atmosphere at 580 □ with time on stream.

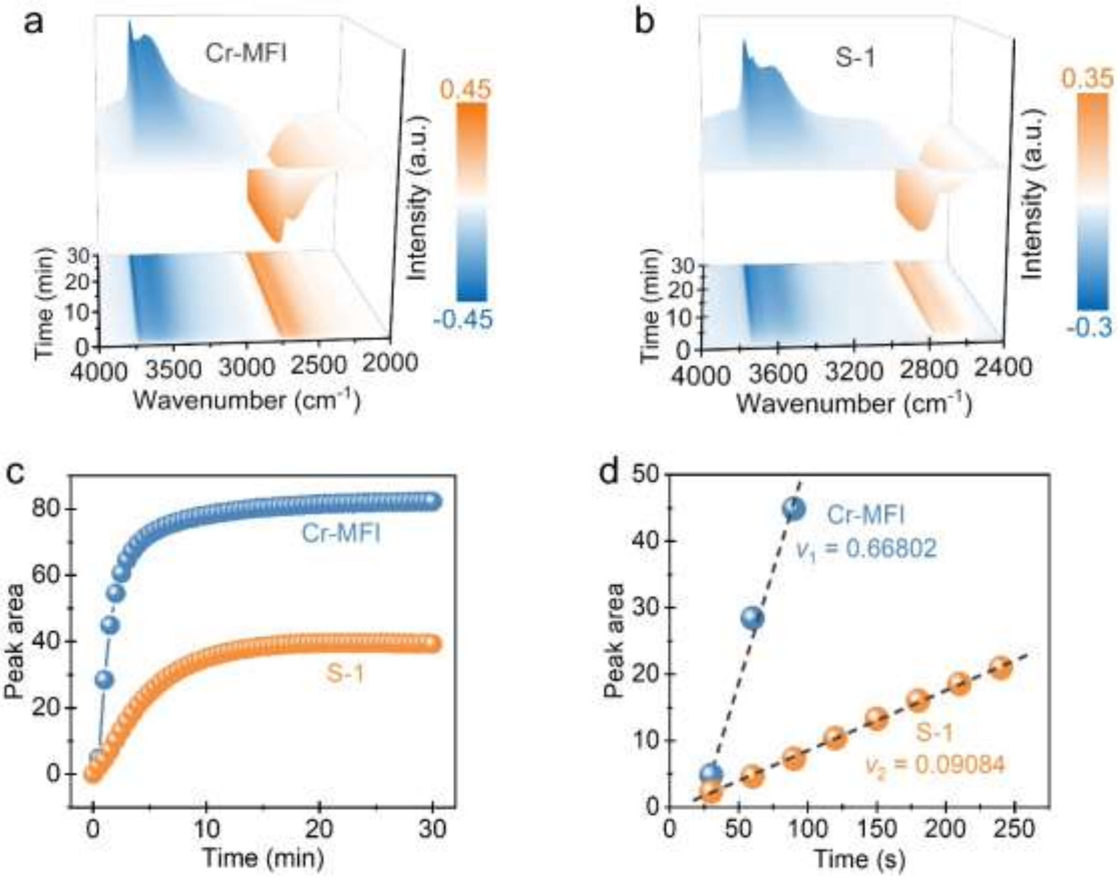

Figure S34. *In situ* FTIR spectroscopy study of Cr-MFI and S-1 pretreated in D_2 atmosphere. (a) D_2 exchange experiment on Cr-MFI. (b) D_2 exchange experiment on S-1. (c) Peak areas of Cr-MFI and S-1 in the range of 2,370~2,800 cm^{-1}. (d) H-D exchange rate of Cr-MFI and S-1.

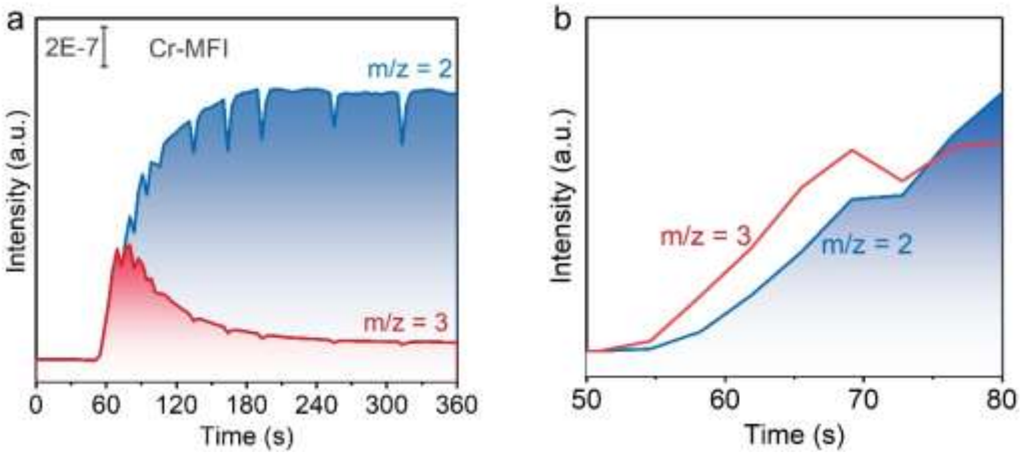

Figure S35. On-line mass spectroscopy (MS) analysis of the generated HD (m/z = 3) and H_2 (m/z = 4) in PDH process over D-labelled Cr-MFI.

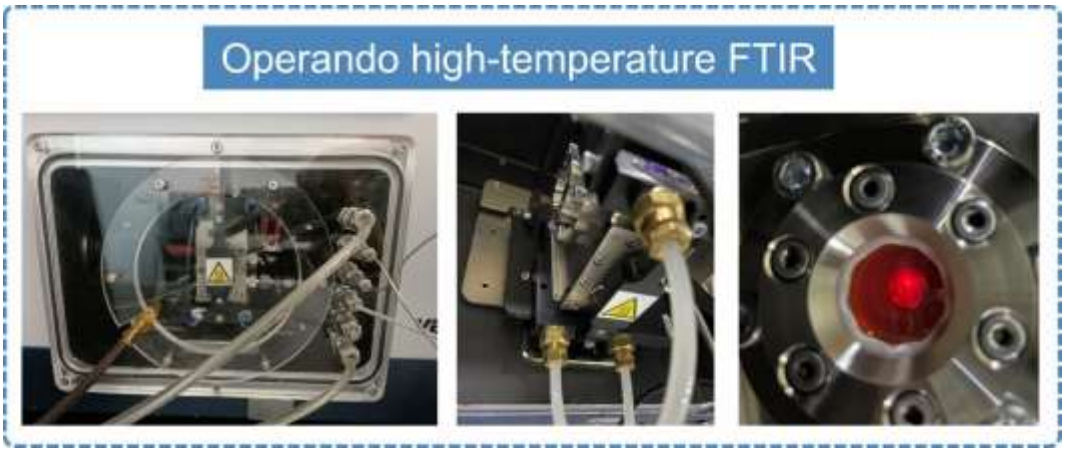

Figure S36. Operando high-temperature FTIR instrument.

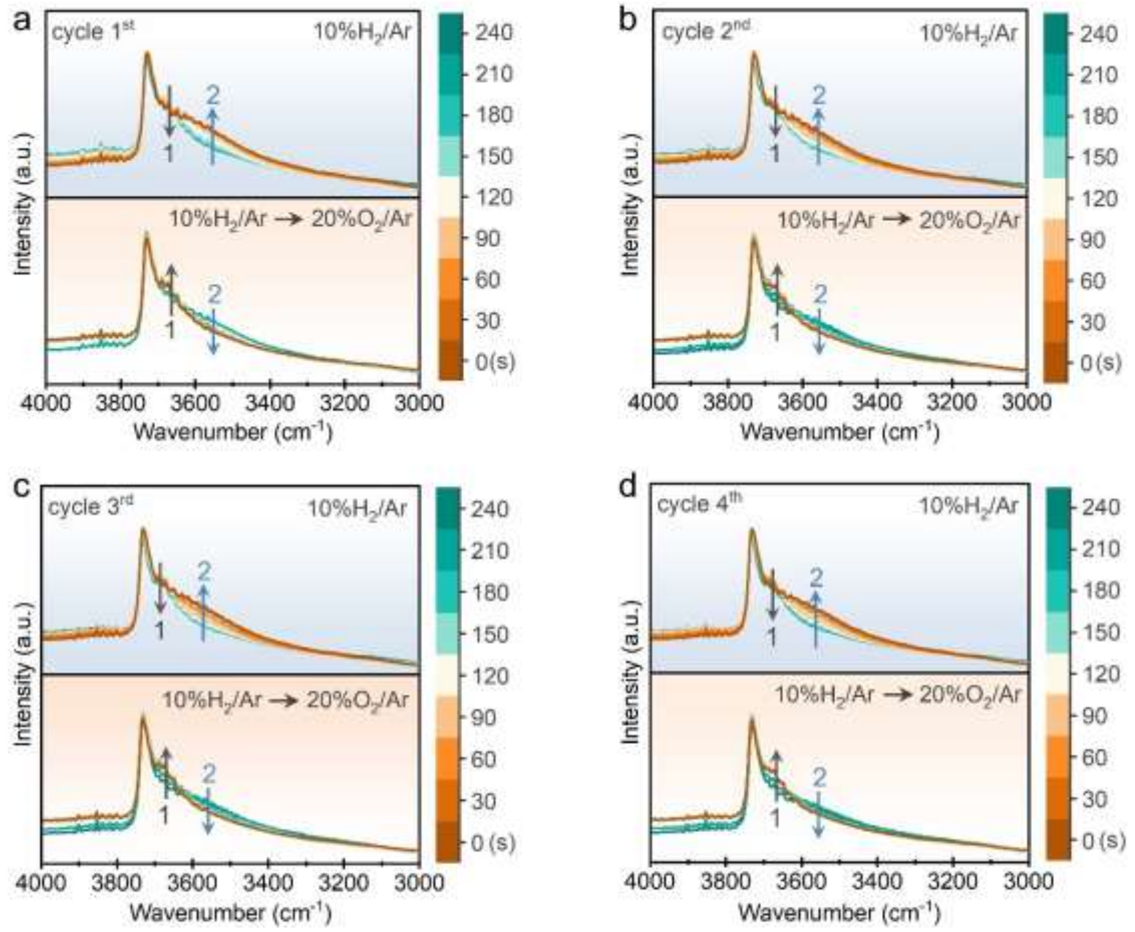

Figure S37. Operando high-temperature FTIR spectra of Cr-MFI at different atmospheres for another four cycles.

3. Tables

Table S1. The physicochemical properties of S-1 and Cr-MFI samples.

Sample	Cr content [a] (wt%)	S_{BET} [b] (m^2 g^{-1})	V_{total} [c] (cm^3 g^{-1})	LAS density [d] (mmol g^{-1})	TOF_{Cal} [e] (h^{-1})
S-1	—	451	0.34	—	—
$Cr_{0.22}$-MFI	0.22	445	0.37	0.006	121.9 ± 20.8
$Cr_{0.72}$-MFI	0.72	449	0.39	0.025	124.3 ± 9.9
$Cr_{1.01}$-MFI	1.01	458	0.33	0.038	125.3 ± 17.4
$Cr_{1.8}$-MFI	1.8	389	0.28	0.045	76.3 ± 0.24

[a] The Cr content was characterized by XRF. [b] Specific surface area obtained by the BET method. [c] Total pore volume was obtained at P/P_0 = 0.97. [d] The LAS densities of Cr-MFI were determined by FTIR spectra (the band at 1450 cm^{-1}) after pyridine adsorption and evacuation at the temperature of 473 K. [e] The calculated TOF_{cal} values were based on all Cr atoms.

Table S2. Structural parameters of Cr-MFI extracted from quantitative EXAFS curve-fitting.

Sample	Shell	N[b]	R (Å)[c]	σ^2 ($Å^2$)[d]	E_0 Cr (eV)	χ^2-like residual (10^{-6})
Cr-MFI[a]	Cr−O_1	3.0	1.946	0.001	6005.5	4.12
	Cr−O_2	1.0	2.547	0.001		
	Cr−Si_1	3.0	3.165	0.015		
	Cr−Si_2	1.0	3.959	0.011		

[a] The data range used for fitting in k-space and R-space are 2.5~11.0 $Å^{-1}$ and 1.0~3.0 Å, respectively. [b] Coordination number, two kinds of Cr−O (3Cr−O_1 and 1Cr−O_2) and Cr−Si (3Cr−Si_1 and 1Cr−Si_2) were fixed based on the calculated structure. [c] Distance between adsorber and backscatter atoms. [d] Debye-Waller factor.

Table S3. Catalytic performance of Cr/SiO_2, Cr/Al_2O_3 and Cr-MFI for PDH at 580 □ under different flow conditions.

Sample	Time (h)	Conversion (%)	Selectivity (%)	WHSV (h^{-1})	k_d[a]
1%Cr/SiO_2	0	1.1	68.4	0.6	
	10	1.0	61.2		
1%Cr/Al_2O_3	0	31.9	95.1	0.6	
	10	10.4	94.8		0.140
$Cr_{0.22}$-MFI	0	18.1	95.2	0.6	
	5	15.0	94.9		0.045
$Cr_{0.72}$-MFI	0	36.8	93.8	0.6	
	5	31.5	94.1		0.047
$Cr_{1.01}$-MFI	0	43.7	93.0	0.6	
	5	36.9	93.5		0.057
	24	28.3	94.0		0.028
	0	24.2	89.4	2.4	
	24	13.3	92.1		0.031
$Cr_{1.8}$-MFI	0	46.3	87.5	0.6	
	5	31.4	92.4		0.127

[a] k_d (h^{-1}) is the deactivation rate.

Table S4. Comparison of the Cr-based catalysts for PDH.

No.	Sample	Cr content (wt%)	Reaction conditions	Conversion (%)	Selectivity (%)	TOF (h^{-1})	Ref.
1	Cr(III)/SiO_2	/	550 □, 25%C_3H_8/Ar, 10 mL min^{-1}, GHSV = 353 h^{-1}, 70 mg catalyst, TOS = 2 h	3	72	5	21
2	Cr(III)/SiO_2-Al_2O_3	0.21		3	70	28	21
3	Cr(III)/Al_2O_3	0.18		3	66	34	21
4	CrO_x/Al_2O_3	1.5		17	88	31	21
5	5Cr-SBA-15	5	580 □, 10%C_3H_8/Ar, 50 mL min^{-1}, 50 mg catalyst, TOS = 20 min	18	80	40	22
6	5Cr-Al_2O_3	5		23	80	51	22
7	S-CrZr_650	4.4	550 □, 40%C_3H_8/N_2, 12 mL min^{-1}, 70 mg catalyst	25	92	49.9	23
8	Cr/Al_2O_3-800	5	580 □, 5%C_3H_8/Ar, 20 mL min^{-1}, WHSV = 0.6 h^{-1}, TOS = 30 min	25.4	96.2	3.4	24
9	Cr20/SBA-15	20	550 □, 6.7%C_3H_8/He, 30 mL min^{-1}, WHSV = 1.2 h^{-1}, TOS = 10 min	25.7	86	1.5	25
10	Cr20/MCM-41			28.9	86	1.7	25
11	Cr20/Al_2O_3-n			33.8	94	2.2	25
12	CrZr5K0.5	8	550 □, 6.7%C_3H_8/He, 30 mL min^{-1}, WHSV = 1.2 h^{-1}, TOS = 40 min	32	96	5.3	26
13	Cr20/AC	20	550 □, 6.7%C_3H_8/He, 30 mL min^{-1}, WHSV = 1.2 h^{-1}, TOS = 10 min	29.9	90.3	1.9	27
14	Cr20/CMK-3	20		47.4	84.7	2.8	27
15	Cr-Al-800	12.8	600 □, C_3H_8 8 mL min^{-1}, 0.1 g catalyst, TOS = 10 min	33.2	90.4	26.1	28
16	Cr-Al-Ref	12.8		40.4	84.7	29.8	28
17	10Cr/MCM-41	10	630 □, 14%C_3H_8/N_2, 29 mL min^{-1}, 0.4 g catalyst, TOS = 30min	40	80	4.5	29
18	3Cr5Sn/Al_2O_3	3	600 □, 14.3%C_3H_8/N_2, 35 mL min^{-1}, 0.4 g catalyst, TOS = 60 min	40	95	22	30
19	Cr/Al_2O_3	1	580 □, 5%C_3H_8/Ar, 20 mL min^{-1}, WHSV = 0.6 h^{-1}, 0.2 g catalyst, TOS = 30 min	34.7	94.9	22.9	This work
20	Cr/SiO_2	1		1.01	65.6	0.5	This work
★	Cr-MFI	1.01	580 □, 5%C_3H_8/Ar, 20 mL min^{-1}, WHSV = 0.6 h^{-1}, 0.2 g catalyst, TOS = 30 min	43.7	93.0	28.3	This work
★	Cr-MFI	1.01	580 □, 10%C_3H_8/Ar, 20 mL min^{-1}, WHSV = 7.86 h^{-1}, 30 mg catalyst, TOS = 30 min	13.6	94.6	118	This work

Table S5. Comparison of the Pt-based catalysts for PDH.

Sample	Pt content (wt%)	Reaction conditions	Conversion (%)	Selectivity (%)	TOF (h^{-1})	Ref.
Pt/Al_2O_3	0.3	600 □, $5\%C_3H_8/N_2$, WHSV = 0.6 h^{-1}, 0.33 g catalyst, TOS = 20 h	/	73	25.2	31
Pt/OLG	0.5	600 □, $5\%C_3H_8/N_2$, WHSV = 0.6 h^{-1}, 0.2 g catalyst, TOS = 20 h	/	81.1	17	31
Pt/ND@G			/	88	~56	31
PtIn/Mg(Al)O-1	0.6	620 □, $H_2:C_3H_8:Ar$ = 7:8:35, WHSV = 3.3 h^{-1}, 0.3 g catalyst	~10	80	138	32
PtIn/Mg(Al)O-2			~57.2	~95	252	32
PtIn/Mg(Al)O-4			~61.3	>94	276	32
PtIn/Mg(Al)O-6			~51	~91	174	32
PtIn/Mg(Al)O-8			~45	~87	162	32
$Pt/AlMgO_x$	0.35	550 □, $H_2:C_3H_8$ = 0.25, 12 mL min^{-1}, WHSV = 8 h^{-1}, TOS = 4 h, 0.5 g catalyst	7	~98	257	33
$Pt/Mg(Zn)AlO_x$-0.1	0.28		20	~99	1273	33
$Pt/Mg(Zn)AlO_x$-0.5	0.33		~13	99	687	33
$Pt/Mg(Zn)AlO_x$-0.7	0.35		~14	~99	775	33
$Pt/AlZnO_x$	0.36		19	99	579	33
Pt/SiO_2	~3	550 □, $25\%C_3H_8/Ar$, 50 mL min^{-1}, $WHSV_{Pt}$ = 834 h^{-1}, TOS = 6 min	17.2	81.5	110	34
$PtMn/SiO_2$	2.97	550 □, $25\%C_3H_8/Ar$, 50 mL min^{-1}, $WHSV_{Pt}$ = 2246 h^{-1}, TOS = 6 min	39.9	96.1	822	34
$PtMn/SiO_2$	2.97	550 □, $25\%C_3H_8/Ar$, 50 mL min^{-1}, $WHSV_{Pt}$ = 1160 h^{-1}, TOS = 6 min	40.7	92.5	416	34
$Pt(0.05)Mn/SiO_2$	0.05	550 □, $25\%C_3H_8/Ar$, 50 mL min^{-1}, $WHSV_{Pt}$ = 27369 h^{-1}, TOS = 6 min	22.2	98.4	5705	34
$Pt(0.05)Mn/SiO_2$	0.05	550 □, $H_2:C_3H_8:Ar$ = 1:1:3, 50 mL min^{-1}, $WHSV_{Pt}$ = 27369 h^{-1}, TOS = 6 min	17.8	97.3	4523	34
Cr-MFI	1.01	580 □, $5\%C_3H_8/Ar$, 20 mL min^{-1}, WHSV = 0.6 h^{-1}, 0.2 g catalyst, TOS = 30 min	43.7	93.0	28.3	This work
Cr-MFI	1.01	580 □, $10\%C_3H_8/Ar$, 20 mL min^{-1}, WHSV = 7.86 h^{-1}, 30 mg catalyst, TOS = 30 min	13.6	94.6	118	This work

Table S6. Comparison of the transition metal-based catalysts in PDH.

Sample	Metal content (wt%)	Reaction conditions	Conversion (%)	Selectivity (%)	TOF (h^{-1})	Ref.
Fe/SiO_2	/	650 □, 3%C_3H_8/Ar, 55 mL min^{-1}, 1 g catalyst	/	99	1~1.5	35
[Fe]ZSM-5(26)	3.4	500 □, 5%C_3H_8/Ar, 80 mL min^{-1}, 1 g catalyst	3.6	85	0.54	36
		530 □, 5%C_3H_8/Ar, 80 mL min^{-1}, 1 g catalyst	7.2	78	1	36
0.5VSiBeta	0.5	600 □, 5%C_3H_8/Ar, 20 mL min^{-1}, 0.2 g catalyst, TOS = 30 min	20.7	93.7	26.5	37
1VSiBeta	1		31.9	93.5	20.4	37
3VSiBeta	3		37.8	88.4	7.6	37
7VSiBeta	7		39.9	89.8	3.5	37
10VSiBeta	10		38.1	91.7	2.4	37
0.5CoSiBeta	0.5	600 □, 5%C_3H_8/Ar, 20 mL min^{-1}, 0.3 g catalyst, TOS = 30 min	53	98.2	54.8	38
1CoSiBeta	1		58.8	98.4	30.5	38
3CoSiBeta	3		80.3	94.6	13.3	38
10CoSiBeta	10		81.4	94.4	4.0	38
Co-MFI	1.12	580 □, 5%C_3H_8/Ar, 20 mL min^{-1}, 0.2 g catalyst, TOS = 30 min	41	97.3	112.7	39
Ni-P/SiO_2		600 □, 5%C_3H_8/N_2, 50 mL min^{-1}, 1 g catalyst	18	90	2.9	40
Znβ-3	3	600 □, 5%C_3H_8/Ar, 20 mL min^{-1}, 0.3 g catalyst, TOS = 30 min	37.9	95.6	7	41
Znβ-7	7		46.3	95.0	3.6	41
Znβ-10	10		53.3	92.9	2.9	41
Znβ-20	20		36.1	94.4	1	41
ZnO_x NP/MCM-41	/	550 □, 40%C_3H_8/N_2, 10 mL min^{-1}, 50 mg catalyst	/	/	2.52	42
Binuclear ZnO_x/S-1_1	/		/	/	239	42
Cr-MFI	1.01	580 □, 5%C_3H_8/Ar, 20 mL min^{-1}, WHSV = 0.6 h^{-1}, 0.2 g catalyst, TOS = 30 min	43.7	93.0	28.3	This work
Cr-MFI	1.01	580 □, 10%C_3H_8/Ar, 20 mL min^{-1}, WHSV = 7.86 h^{-1}, 30 mg catalyst, TOS = 30 min	13.6	94.6	118	This work

Table S7. The distances of Cr−C, Cr···O, Cr−O, and Cr−H during PDH process on Cr-MFI zeolite with Cr at T_3 site with whole microenvironment.

	d(Cr−C) Å	*d*(Cr…$OH^{\delta-}$) Å	*d*(Cr−O) Å	*d*(Cr−$H^{\delta+}$) Å
I	–	2.404	1.8, 1.806, 1.809	–
II	3.44	2.398	1.8, 1.803, 1.808	–
TS1	2.223	2.274	1.839, 1.871, 2.04	–
III	2.022	2.261, 2.939	1.852, 1.867	–
TS2	1.998, 2.191	2.746, 3.501	1.950, 2.054	–
IV	2.265, 2.324	2.717, 3.566	2.116, 2.281	–
V	–	2.379, 2.675, 3.437	2.008	–
TS3	–	2.172, 2.454, 3.148	1.949	–
VI	–	2.198, 2.804	1.840, 1.864	1.601
TS4	–	2.061, 2.154	1.838, 1.853	1.720
VII	–	2.041	1.844, 1.859, 1.92	–

Table S8. The distances of Cr−C, Cr···O, Cr−O, and Cr−H during PDH process on Cr-MFI zeolite with Cr at T_3 site without whole microenvironment.

	d(Cr−C) Å	*d*(Cr…OH$^{\delta-}$) Å	*d*(Cr−O) Å	*d*(Cr−H$^{\delta+}$) Å
I	–	2.031	1.765, 1.77, 1.77	–
II	3.485	2.466	1.799, 1.817, 1.825	–
TS1	2.209	2.866	1.828, 1.857, 2.038	–
III	2.208	2.178, 3.14	1.849, 1.904	–
TS2	3.463	3.157, 4.013	1.959, 2.131	–
IV	2.247	2.351, 2.868, 3.474	2.074	–
V	–	2.182, 2.753, 2.915	1.915	–
TS3	–	2.220, 2.587	1.900, 2.041	1.744
VI	–	2.168, 2.372	1.840, 1.887	1.606
TS4	–	2.031, 2.294	1.824, 1.836	1.681
VII	–	2.495	1.799, 1.818, 1.823	–

4. References

(1) Emeis, C. A. Determination of integrated molar extinction coefficients for infrared absorption bands of pyridine adsorbed on solid acid catalysts. *J. Catal.* **141**, 347−354 (1993).

(2) Perdew, J. P., Burke, K., and Ernzerhof, M. Generalized gradient approximation made simple. *Phys. Rev. Lett.* **77**, 3865−3868 (1993).

(3) Kresse, G., and Hafner, J. Ab initio molecular dynamics for liquid metals. *Phys. Rev. B* **47**, 558−561 (1993).

(4) Kresse, G., and Hafner, J. Ab initio molecular-dynamics simulation of the liquid-metal-amorphous-semiconductor transition in germanium. *Phys. Rev. B* **49**, 14251−14269 (1994).

(5) Kresse, G., and Furthmüller, J. Efficient iterative schemes for ab initio total-energy calculations using a plane-wave basis set. *Phys. Rev. B* **54**, 11169−11186 (1996).

(6) Kresse, G., and Furthmüller, J. Efficiency of ab-initio total energy calculations for metals and semiconductors using a plane-wave basis set. *Comput. Mater. Sci.* **6**, 15−50 (1996).

(7) Grimme, S., Ehrlich, S., and Goerigk, L. Effect of the damping function in dispersion corrected density functional theory. *J. Comput. Chem.* **32**, 1456−1465 (2011).

(8) Hammer, B., Hansen, L. B., and Nørskov, J. K. Improved adsorption energetics within density-functional theory using revised Perdew-Burke-Ernzerhof functionals. *Phys. Rev. B* **59**, 7413−7421 (1999).

(9) Blöchl, P. E. Projector augmented-wave method. *Phys. Rev. B* **50**, 17953−17979 (1994).

(10) Henkelman, G., Uberuaga, B. P., and Jónsson, H. A climbing image nudged elastic band method for finding saddle points and minimum energy paths. *J. Chem. Phys.* **113**, 9901−9904 (2000).

(11) Monkhorst, H. J.; Pack, J. D. Special points for Brillouin-zone integrations. *Phys. Rev. B* **13**, 5188– 5192 (1976).

(12) Wechuysen, B. M.; Wachs, I. E.; Schoonhedyt, R. A. Surface chemistry and spectroscopy of chromium in inorganic oxides. *Chem. Rev.* **96**, 3327−3349 (1996).

(13) Baek, J.; Yun, H. J.; Yun, D.; Choi, Y.; Yi, J. Preparation of highly dispersed chromium oxide catalysts supported on mesoporous silica for the oxidative dehydrogenation of propane using CO_2: Insight into the nature of catalytically active chromium sites. *ACS Catal.* **2**, 1893−1903 (2012).

(14) Gao, J.; Zheng, Y.; Tang, Y.; Jehng, J. M.; Grybos, R.; Handzlik, J.; Wachs, I. E.; Podkolzin, S. G. Spectroscopic and computational study of Cr oxide structures and their anchoring sites on ZSM□5 zeolites. *ACS Catal.* **5**, 3078−3092 (2015).

(15) Zheng, A.; Liu, S. B.; Deng, F. ^{31}P NMR chemical shifts of phosphorus probes as reliable and practical acidity scales for solid and liquid catalysts. *Chem. Rev.* **117**, 12475−12531 (2017).

(16) Dubray, F.; Moldovan, S.; Kouvatas, C.; Grand, J.; Aquino, C.; Barrier, N.; Gilson, J. P.; Nesterenko, N.; Minoux, D.; Mintova, S. Direct evidence for single molybdenum atoms incorporated in the framework of MFI

zeolite nanocrystals. *J. Am. Chem. Soc.* **141**, 8689−8693 (2019).

(17) Yi, X.; Chen, W.; Xiao, Y.; Liu, F.; Yu, X.; Zheng, A. Spectroscopically visualizing the evolution of hydrogen-bonding interactions. *J. Am. Chem. Soc.* **145**, 27471−27479 (2023).

(18) Abidi, N.; Boudjema, Y.; Rivallan, M.; Chizallet, C.; Larmier, K. Challenging the distinction between "open" and "closed" Sn sites in β zeolite by deuterated acetonitrile adsorption: Experimental and theoretical insights. *J. Phys. Chem. C* **129**, 14011−14019 (2025).

(19) Alghannam, A.; Bell, A. T. Effects of cofeeding hydrogen on propane dehydrogenation catalyzed by isolated iron sites incorporated into dealuminated BEA. *J. Am. Chem. Soc.* **147**, 1677−1693 (2025).

(20) Yuan, Y.; Brady, C.; Annamalai, L.; Lobo, R. F.; Xu, B. Ga speciation in Ga/H-ZSM-5 by in-situ transmission FTIR spectroscopy. *J. Catal.* **393**, 60−69 (2021).

(21) Delley, M. F.; Silaghi, M. C.; Nuñez-Zarur, F.; Kovtunov, K. V.; Salnikov, O. G.; Estes, D. P.; Koptyug, I. V.; Comas-Vives, A.; Copéret, C. X−H bond activation on Cr(III),O sites (X = R, H): Key steps in dehydrogenation and hydrogenation processes. *Organometallics* **36**, 234−244 (2017).

(22) Kumar, M. S.; Hammer, N.; Rønning, M.; Holmen, A.; Chen, D.; Walmsley, J. C.; Øye, G. The nature of active chromium species in Cr-catalysts for dehydrogenation of propane: New insights by a comprehensive spectroscopic study. *J. Catal.* **261**, 116−128 (2009).

(23) Han, S.; Otroshchenko, T.; Zhao, D.; Lund, H.; Rockstroh, N.; Vuong, T. H.; Rabeah, J.; Rodemerck, U.; Linke, D.; Gao, M.; Jiang, G.; Kondratenko, E. V. The effect of ZrO_2 crystallinity in $CrZrO_x/SiO_2$ on non-oxidative propane dehydrogenation. *Appl. Catal. A* **590**, 117350 (2020).

(24) Hu, Z. P.; Wang, Z.; Yuan, Z. Y. Cr/Al_2O_3 catalysts with strong metal-support interactions for stable catalytic dehydrogenation of propane to propylene. *Mol. Catal.* **493**, 111052 (2020).

(25) Węgrzyniak, A.; Jarczewski, S.; Węgrzynowicz, A.; Michorczyk, B.; Kuśtrowski, P.; Michorczyk, P. Catalytic behavior of chromium oxide supported on nanocasting-prepared mesoporous alumina in dehydrogenation of propane. *Nanomaterials* **7**, 249 (2017).

(26) Węgrzyniak, A.; Rokicińska, A.; Hędrzak, E.; Michorczyk, B.; Zeńczak-Tomera, K.; Kuśtrowski, P.; Michorczyk, P. High-performance Cr−Zr−O and Cr−Zr−K−O catalysts prepared by nanocasting for dehydrogenation of propane to propene. *Catal. Sci. Technol.* **7**, 6059–6068 (2017).

(27) Węgrzyniak, A.; Jarczewski, S.; Wach, A.; Hędrzak, E.; Kuśtrowski, P.; Michorczyk, P. Catalytic behaviour of chromium oxide supported on CMK-3 carbon replica in the dehydrogenation propane to propene. *Appl. Catal. A* **508**, 1−9 (2015).

(28) Gao, X. Q.; Lu, W. D.; Hu, S. Z.; Li, W. C.; Lu, A. H. Rod-shaped porous alumina-supported Cr_2O_3 catalyst with low acidity for propane dehydrogenation. *Chin. J. Catal.* **40**, 184−191 (2019).

(29) He, D.; Zhang, Y.; Yang, S.; Mei, Y.; Luo, Y. Investigation of the isolated Cr(VI) species in Cr/MCM-41 catalysts and its effect on catalytic activity for dehydrogenation of propane. *ChemCatChem* **10**, 5434−5440 (2018).

(30) Zhang, L.; Chen, K.; Chen, H.; Han, X.; Liu, C.; Qiao, L.; Wu, W.; Yang, B. Elucidating the promoting advantages and fundamentals for their creation in Sn-modified commercial CrO_x/Al_2O_3 catalyst for propane

dehydrogenation. *Chem. Eng. J.* **483**, 149366 (2024).
(31) Liu, J.; Yue, Y.; Liu, H.; Da, Z.; Liu, C.; Ma, A.; Rong, J.; Su, D.; Bao, X.; Zheng, H. Origin of the robust catalytic performance of nanodiamond–graphene-supported Pt nanoparticles used in the propane dehydrogenation reaction. *ACS Catal.* **7**, 3349−3355 (2017).
(32) Xia, K.; Lang, W. Z.; Li, P. P.; Long, L. L.; Yan, X.; Guo, Y. J. The influences of Mg/Al molar ratio on the properties of PtIn/Mg(Al)O_x catalysts for propane dehydrogenation reaction. *Chem. Eng. J.* **284**, 1068−1079 (2016).
(33) Belskaya, O. B., Stepanova, L. N., Gulyaeva, T. I., Erenburg, S. B., Trubina, S. V., Kvashnina, K., Nizovskii, A. I.; Kalinkin, A. V.; Zaikovskii, V. I.; Bukhtiyarov, V. I.; Likholobov, V. A. Zinc influence on the formation and properties of Pt/Mg(Zn)AlO_x catalysts synthesized from layered hydroxides. *J. Catal.* **341**, 13−23 (2016).
(34) Rochlitz, L.; Pessemesse, Q.; Fischer, J. W.; Klose, D.; Clark, A. H.; Plodinec, M.; Jeschke, G.; Payard, P. A.; Copéret, C. A robust and efficient propane dehydrogenation catalyst from unexpectedly segregated Pt_2Mn nanoparticles. *J. Am. Chem. Soc.* 144, 13384−13393 (2022).
(35) Hu, B.; Schweitzer, N. M.; Zhang, G.; Kraft, S. J.; Childers, D. J.; Lanci, M. P.; Miller, J. T.; Hock, A. S. Isolated Fe^{II} on silica as a selective propane dehydrogenation catalyst. *ACS Catal.* **5**, 3494−3503 (2015).
(36) Yun, J. H.; Lobo, R. F. Catalytic dehydrogenation of propane over iron-silicate zeolites. *J. Catal.* 312, 263−270 (2014).
(37) Chen, C.; Sun, M.; Hu, Z.; Liu, Y.; Zhang, S.; Yuan, Z. Y. Nature of active phase of VO_x catalysts supported on SiBeta for direct dehydrogenation of propane to propylene. *Chin. J. Catal.* **41**, 276−285 (2020).
(38) Chen, C.; Zhang, S.; Wang, Z.; Yuan, Z. Y. Ultrasmall Co confined in the silanols of dealuminated beta zeolite: A highly active and selective catalyst for direct dehydrogenation of propane to propylene. *J. Catal.* **383**, 77−87 (2020).
(39) Hu, Z. P.; Qin, G.; Han, J.; Zhang, W.; Wang, N.; Zheng, Y.; Wang, N.; Zheng, Y.; Jiang, Q.; Ji, T.; Yuan, Z. Y.; Xiao, J.; Wei, Y.; Liu, Z. Atomic insight into the local structure and microenvironment of isolated Co-motifs in MFI zeolite frameworks for propane dehydrogenation. *J. Am. Chem. Soc.* **144**, 12127−12137 (2022).
(40) Zhang, G.; Yang, C.; Miller, J. T. Tetrahedral nickel(II) phosphosilicate single-site selective propane dehydrogenation catalyst. *ChemCatChem* **10**, 961−964 (2018).
(41) Chen, C.; Hu, Z.; Ren, J.; Zhang, S.; Wang, Z.; Yuan, Z. Y. ZnO nanoclusters supported on dealuminated zeolite β as a novel catalyst for direct dehydrogenation of propane to propylene. *ChemCatChem* **11**, 868−877 (2019).
(42) Zhao, D.; Tian, X.; Doronkin, D. E.; Han, S.; Kondratenko, V. A.; Grunwaldt, J. D.; Perechodjuk, A.; Vuong, T. H.; Rabeah, J.; Eckelt, R.; Rodemerck, U.; Linke, D.; Jiang, G.; Jiao, H.; Kondratenko, E. V. In situ formation of ZnO_x species for efficient propane dehydrogenation. *Nature* **599**, 234−238 (2021).
